# Awareness, facilitators, barriers, and behaviours surrounding brain health: a large-scale cross-sectional survey of adults across UK and Ireland

**DOI:** 10.1186/s12889-025-24175-0

**Published:** 2025-10-01

**Authors:** R. F. Townsend, O. M. Shannon, E. Stevenson, C. Ritchie, A. M. Minihane, P. Devine, S. Casey, N. Fullerton, I. Leroi, B. Lawlor, R. O’Sullivan, B. McGuinness, J. V. Woodside, C. T. McEvoy

**Affiliations:** 1 Centre for Public Health, Queen’s University Belfast, Belfast, Northern Ireland; 2 School of Biomedical, Nutritional and Sport Sciences, Faculty of Medical Sciences, Newcastle University, Newcastle upon Tyne, UK; 3 Human Nutrition & Exercise Research Centre, Population Health Sciences Institute, Newcastle University, Newcastle upon Tyne, UK; 4 Department of Medicine, University of St. Andrews, St Andrews, Scotland, UK; 5 Scottish Brain Sciences, Edinburgh, Scotland, UK; 6 Nutrition and Preventive Medicine Group, Norwich Medical School, University of East Anglia, Norwich, UK; 7 School of Social Sciences, Education and Social Work, Queen’s University Belfast, Belfast, Northern Ireland; 8 Age NI , Belfast, Northern Ireland; 9 Brain Health Scotland, Edinburgh, Scotland, UK; 10 Global Brain Health Institute, Trinity College Dublin, Dublin, Ireland; 11 Institute of Public Health, Dublin, Ireland; 12 Bamford Centre for Mental Health and Well Being, Ulster University, Belfast, Northern Ireland

**Keywords:** Brain health, Lifestyle, Dementia risk reduction, Health promotion, Cross-sectional, Survey

## Abstract

**Background:**

Almost half of all dementia cases could theoretically be delayed or prevented by addressing risk factors at the population level. However, dementia risk reduction requires awareness of, and action on, modifiable risk factors. This study aimed to explore public awareness of brain health, and the facilitators for, and barriers to, engaging in preventative action to reduce dementia risk, across the UK and Ireland.

**Methods:**

The Brain Health and Lifestyle Survey (BHLS) was a co-developed and evidence-informed online survey, underpinned by behaviour change frameworks. The BHLS was distributed via convenience sampling to individuals aged ≥ 40 years living in the UK and Ireland. It comprised 31 main questions on awareness, beliefs and behaviour change surrounding brain health and took approximately 20–25 min to complete. Ethical approval was obtained from Queen’s University Belfast [Ref: MHLS20_162].

**Results:**

A total of 6816 respondents (75% UK; 25% Ireland) completed the BHLS between February and June 2021. Most respondents were aged 50–74 years (78%), female (79%), white (99%), overweight (59%) and highly educated (64%). The majority of respondents rated their brain health as good (79%) and there was high awareness of protective factors, including cognitively stimulating activities (91%) and physical exercise (88%). However, awareness of risk factors such as hypertension (62%), midlife obesity (61%), air pollution (50%) and hearing loss (35%) was lower. Awareness differed according to demographic factors, with lower awareness among respondents aged 40–49 years, and those with lower educational attainment. The identified barriers to adopting a brain-healthy lifestyle were implementing changes which were not enjoyable (44%), lack of self-motivation (33%), and a lack of trusted information (27%). Facilitators for adopting a brain-healthy lifestyle included: noticing problems with brain health (70%) and receiving personalised advice (51%).

**Conclusion:**

Understanding of brain health and dementia risk reduction was variable in this large sample of UK and Irish citizens. There were identified gaps in awareness of risk factors relating to cardiometabolic health, hearing loss, and air pollution. These findings highlight the need for credible sources of accessible and relevant information to improve awareness and behaviours surrounding brain health.

**Supplementary Information:**

The online version contains supplementary material available at 10.1186/s12889-025-24175-0.

## Introduction

Dementia is one of the leading causes of morbidity and disability in older age. Over 1 million individuals live with dementia across the UK and Ireland; this figure is projected to double by 2040 in line with population ageing [1]. There is currently no cure for dementia, however it has been estimated that up to 45% of future dementia cases in the population could be delayed or prevented by addressing 14 modifiable risk factors across the life course [2]. The World Health Organisation (WHO) Public Health Response to Dementia Report [3] has previously recommended the implementation of campaigns to increase awareness of dementia risk reduction globally. However, it is critical to understand current population dementia literacy, to inform future interventions for dementia risk reduction across the UK and Ireland. Given that mid-life is increasingly recognised as a pivotal period for the implementation of interventions to reduce risk of dementia in later life [4], focusing on individuals aged 40 years and above, may be particularly relevant.

Globally, systematic review data suggest a lack of public awareness of the potential to reduce dementia risk, highlighting themes of fear and stigma surrounding the condition [5, 6]. It has been acknowledged that shifting terminology from dementia-focused, toward the promotion of brain health may be important when discussing dementia risk reduction. For example, results from a longitudinal survey conducted by Alzheimer’s Research UK, suggested only 34% of UK adults believed it was possible to reduce dementia risk, but in contrast 69% felt it was possible to influence their brain health [7]. Chen et al. define “brain health” as a life-long, dynamic state that integrates cognitive, emotional, and motor functions, supported by physiological processes and influenced by biological, psychological, and social factors. This concept emphasizes measurable, subjective aspects, informing policy and care approaches to enhance brain health across the lifespan [8]. To date, studies exploring public understanding of brain health have been conducted internationally, including in Europe, Australia, and the United States, though fewer have focused specifically on public perceptions within the UK and Ireland [9–13].

In UK and Irish populations, previous evidence has suggested that, although some members of the public may believe it is possible to positively influence their brain health, many are uncertain about what specific actions to take [7, 14–17]. Prior studies have often not explored engagement in risk and protective behaviours, simultaneous with awareness and beliefs [7, 15, 16]. Similarly, few have drawn upon behaviour change theory, impeding the ability to identify potential levers for future behaviour change to promote brain health [18].

An exception to this, is the recent Five Lives Brain Health Ireland Survey (FLBHIS) conducted in February 2022, which investigated awareness and barriers surrounding brain health [19, 20]. Results suggested head injury, low mental stimulation, and alcohol consumption were commonly recognised modifiable factors, while a lack of motivation, practical factors, and emotional factors were often cited as barriers. Overall, disparities in awareness and behaviours were evident between demographic groups. For example, individuals with higher educational attainment often had greater awareness of, and lower exposure to risk factors. However, the FLBHIS did not utilise validated questionnaires to assess behaviours related to modifiable factors and focused exclusively on Irish adults (aged ≥ 50 years).

Ultimately, previous evidence has suggested there is fear, stigma and common misconceptions about dementia and its prevention, all of which may prevent individuals from taking preventative action [5, 6]. This is especially important during mid-life (aged ≥ 40 years), and the beginning of the prodromal stage, when several factors may begin to impact brain health and risk of dementia [3, 7, 21]. To address this paucity in the literature, the Brain Health and Lifestyle Survey (BHLS) was designed with adults aged ≥ 40 years old living across the UK and Ireland and underpinned by behaviour change frameworks. The main aims of the BHLS were to (i) evaluate population awareness and beliefs about brain health and modifiable factors for dementia risk reduction; (ii) explore facilitators and barriers for behaviour change to promote brain health; (iii) evaluate engagement in behaviours relevant for dementia risk reduction. In relation to aim (i), the survey specifically assessed awareness of 12 modifiable risk or protective factors which have been identified in WHO and/or previous Lancet Commission. This included: eating a healthy diet, engaging in regular physical activity, socialising, participating in cognitively stimulating activities, avoiding smoking and heavy alcohol consumption, managing mental health, keeping blood pressure under control, maintaining a healthy weight, getting sufficient sleep, using hearing aids to address hearing loss, and avoiding air pollution.

## Materials and methods

### Study design and participants

This study employed a cross-sectional design, consisting of an online survey (Qualtrics XM, 2019). Eligible individuals were aged ≥ 40 years old and lived (or had settled status) in the UK or Ireland. Participants were required to have the ability to understand the English language, the capability to provide consent, and access to an online device. Ethical approval was granted by the Faculty of Medicine, Health and Life Sciences Ethics Committee, Queen’s University Belfast [Ref: MHLS20_162]. All methods were performed in accordance with the Declaration of Helsinki. The survey was distributed from February until July 2021 (4 months) using convenience sampling using a two-step approach. This involved a four-week Facebook (https://en-gb.facebook.com/) advertising campaign, followed by a snowballing approach for promotion across other Join Dementia Research (JDR) and academic, community and charity sector networks. Advertisements included a link to the online survey, which included the participant information sheet on the first page. Informed consent was then obtained electronically, as all participants were required to confirm their consent prior to proceeding with the survey.

### Development of the BHLS

**Fig. 1:**
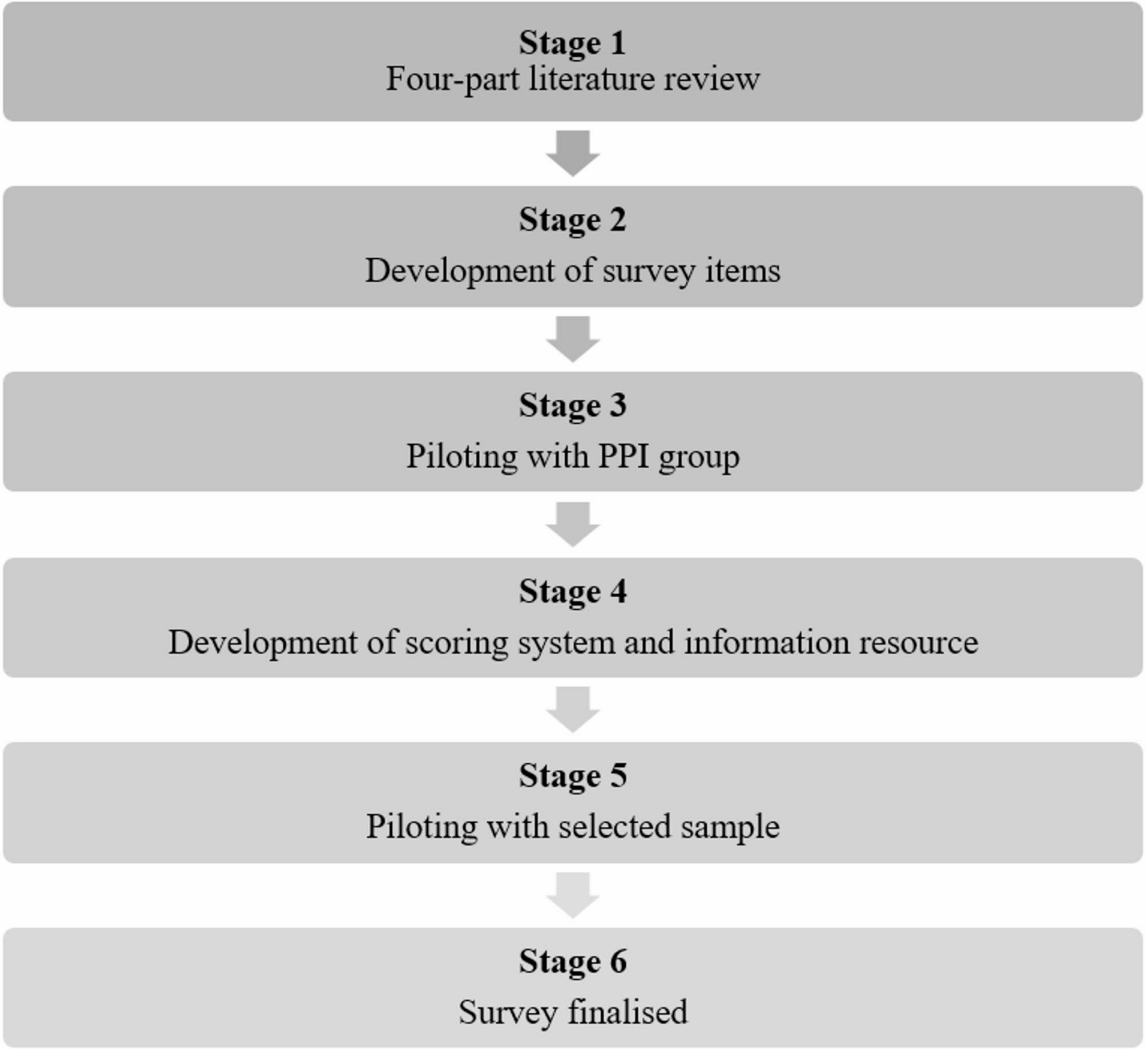
Overview of the development of the BHLS

The BHLS was developed across six stages as outlined in Fig. 1. Stage one involved a rapid scoping review of existing literature (primary and review data) surrounding public awareness of dementia risk reduction [5, 6]. Stage two involved creating or adapting questions (items) to capture data on awareness, beliefs, behaviours, and facilitators/barriers to brain health in the context of dementia risk reduction. Items which assessed awareness of brain health were adopted from previous evidence [14] and revised to ensure answers aligned with evidence from the Lancet Commission Report [22]. Identified survey items were mapped onto the Theoretical Domain Framework (TDF) and Health Beliefs Model (HBM) behaviour change frameworks [23, 24]. Stage three involved piloting the BHLS with the Age Northern Ireland (NI) Consultative Forum, who provided personal and public involvement (PPI) input for the survey design. Feedback from the PPI group led to stage four, the refinement of survey items and the development of a respondent ‘brain healthy lifestyle’ score and information resource for those completing the BHLS. In stage five, the refined survey was piloted again with a convenience sample of 12 staff and students from Queen’s University Belfast, to ensure understanding and technical functionality. The feedback was positive overall, with some constructive comments (i.e., the addition of abstinence from alcohol in potential options), which were addressed in stage six to finalise the survey. The full version of the survey is provided in Supplementary File 1.

### The brain health and lifestyle survey (BHLS)

The BHLS included 31 main questions, with an estimated completion time of 20–25 min (see Supplementary File 1). Survey items related to key target areas: awareness and beliefs surrounding brain health, dementia, and modifiable factors, current engagement in relevant risk and protective behaviours, and the likelihood of future behaviour change for dementia risk reduction.

### BHLS measures

The BHLS captured respondents’ demographic characteristics, such as age, country of residence, gender, education level and ethnicity. Weight status was assessed using a self-perception question to reduce survey burden and capture respondents’ subjective views, which are relevant for understanding behavioural influences. Information on health status was also collected, such as diagnosis of chronic conditions/illnesses, and self-rated health. Awareness surrounding potentially modifiable factors for brain health was quantified using questions adapted from a prior survey in Scotland among younger adults [15]. In this study, the term “awareness” referred to the correct identification of risk and protective factors presented in the survey. Beliefs toward brain health were assessed through questions from the MOCHAD-10 [25], purposefully adapted for use in the BHLS. To capture insight into influences on behaviour change to brain healthy lifestyle respondents were presented with a list of facilitators and barriers and asked to select those which were most important to them. All facilitators and barriers were mapped onto the TDF framework [23], provided in the Supplementary File 2 (Table S1). Respondents were asked to state whether they had previously taken action to protect their brain health. A range of risk and protective behaviours were then displayed, and the respondent was asked to state how frequently they engage in each, this question was adapted from Budin-Ljøsne, Friedman [14]. Respondents were also asked to report their daily/weekly intake of specific foods servings to determine adherence to the Mediterranean-DASH diet intervention for neurodegenerative delay (MIND) diet score [26]. The score ranged from 0 to 15, whereby a higher score indicated greater adherence to the MIND diet. The General Practitioners Physical Activity Questionnaire (GP-PAQ) was included to evaluate physical activity behaviours [27]. Based on responses to the GP-PAQ, respondents were categorised into either active, moderately active, moderately inactive, or inactive [28]. The 4-item Perceived Stress Scale (PSS-4) was included in the survey [29]. Responses to PSS-4 questions were summed to create a score which ranged from 0 to 16, whereby a higher score was indicative of greater psychological global stress.

### Statistical analysis

Data entry, cleaning and analysis were conducted using IBM SPSS (Version 25.0). All collected responses for each question were analysed, including those from respondents who terminated the survey prior to full completion. When examining differences between demographic groups, responses to specific questions were dichotomised or collapsed to increase the sample size per group and maximise statistical power. Education level was collapsed into four categories as degree level and above degree level were merged. Due to the small number of respondents in Wales (*n* = 151), data were merged with respondents who lived in England for analysis (England and Wales). Previous preventative action was dichotomised into yes (‘Yes’) or no (‘No’, ‘I don’t know’). Awareness of modifiable factors was categorised into fully aware (‘I definitely knew this’) and not fully aware (‘I think I knew this’, ‘I did not know this, but it is unsurprising’, ‘I did not know this, and I find it a little surprising’). Beliefs surrounding brain health were categorised into agree (‘Strongly Agree’ and ‘Agree’), not sure (‘Neither agree nor disagree’), and disagree (‘Strongly Disagree’ and ‘Disagree’).

Descriptive statistics (frequency counts (n) and percentages (%)) were used to examine the distribution of responses to survey questions for awareness of modifiable factors, beliefs surrounding brain health, facilitators for, and barriers to behaviour change, and current engagement in risk and protective behaviours (frequency scale questions and GP-PAQ categorisation). To examine differences in the distribution of responses to questions between categories of age, education, and country of residence, chi-squared (χ^2^) tests were conducted, with effect sizes reported using Cramer’s V to assess the strength of associations. Post-hoc tests included Bonferroni corrections for multiple comparisons where applicable. To examine potential differences in MIND and PSS-4 scores between categories of age, education, and country of residence, one-way analyses of variance (ANOVAs) were conducted, with Tukey post-hoc tests (P-values adjusted for multiple comparisons). Descriptive statistics for MIND and PSS-4 scores included means ± SD. Skewed variables were examined using Welch’s ANOVA with Games-Howell post-hoc tests. Effect sizes for ANOVA were reported using partial eta squared (η²). For all analyses, statistical significance was defined as *p* < 0.05.

## Results

In total, 9,127 survey entries were collected. From this, 2,226 responses were removed as no progress was made past the information sheet, including no provision of consent. From the remaining responses, a total of 77 did not meet the eligibility criteria. Reasons for exclusion included ineligible age (*n* = 52) and country (*n* = 25). Seven responses were excluded from the dataset, six due to internal test submissions using the survey preview link during development, and one to a suspicious IP address flagged by Qualtrics’ built-in fraud detection tools. This resulted in a total of 6,816 responses, with 5942 (87%) respondents providing complete data for all survey questions. Missing data were left as system-missing and not imputed. All available data were included in descriptive analyses and inferential analyses (e.g., chi-square tests, ANOVA) were based on complete case data for the relevant variables.

### Respondent demographics

The demographic characteristics of study respondents are outlined in Table 1. Half of the sample lived in England (50%) and a quarter (25%) lived in the Republic of Ireland. Most respondents in the sample were aged 50–74 years (78%), were female (79%), and identified as white (99%). A large proportion had a high level of educational attainment (64% educated to degree level or above), and over half the sample (59%) self-reported as overweight. Most respondents rated their overall health (85%) and brain health (79%) as good. There were significant associations between country and key demographic variables, including gender, educational attainment, and age (all *p* < 0.001). These differences were important to consider when interpreting comparisons between countries, as demographic characteristics may confound observed patterns in awareness, beliefs and behaviours. For example, England and Wales had a higher proportion of older adults and individuals with degree-level education, while the Republic of Ireland had a younger population with lower educational attainment. An overview of the relationship between respondents demographic categories (i.e., country and education, country and age and age and education) are provided in Supplementary File 2 (Figure S1–S3).

### Beliefs surrounding brain health

A total of 40% (*n* = 2422) of the sample agreed they were likely to experience poor brain health in the future. Furthermore, 57% (*n* = 3500) believed there was a strong possibility that their brain health would decline in the next ten years. Nearly three-quarters (72%, *n* = 4424) of respondents stated they would feel differently about themselves if their brain health declined, and the majority (87%, *n* = 5302) acknowledged that they feared the thought of this. There were significant differences in brain health beliefs between demographic categories, details of which, are provided in Supplementary File 2 (Tables S2–S4). Overall, older respondents (aged ≥ 66) and those from England/Wales were more likely to believe there was a greater likelihood of their brain health declining in the next ten years. In contrast, younger respondents (aged 40–49), and those educated to primary level or below were more likely to express anxiety surrounding decline in brain health.

### Awareness of brain health, dementia, and Understanding of modifiable factors

**Fig. 2:**
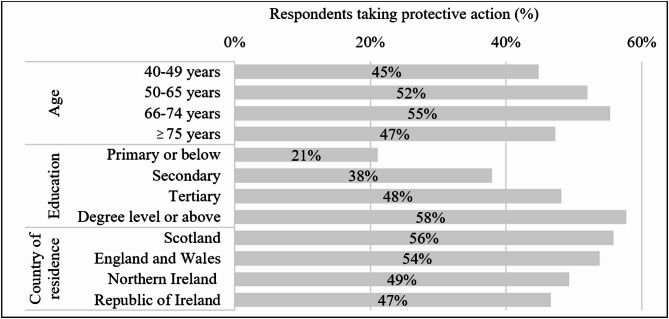
Percentage of participants who reported taking previous protective action by age, education level, and country

One third of respondents (33%; *n* = 2181) thought about their brain health often, while 23% (*n* = 1520) rarely or never thought about their brain health. Respondents who thought about their brain health less, were often more likely to have lower educational attainment and reside across the island of Ireland compared to England (*p* < 0.001). Over half (52%; *n* = 3430) of respondents had taken previous action to protect their brain health. Individuals aged between 40 and 49 years, those with lower educational attainment, and those living in the Republic of Ireland had the least reported preventative action (*p* < 0.001). An overview of the proportion of respondents reporting previous protective action by age, education level, and country is provided in Fig. 2, effect sizes (Cramer’s V) for which, ranged from 0.07 to 0.18.

In relation to dementia literacy, most respondents (92%; *n* = 5494) recognised there are different sub-types of dementia, however 40% (*n* = 2424) believed dementia was another term for Alzheimer’s Disease. Most (94%; *n* = 5639) acknowledged dementia is a disease of the brain, however 29% (*n* = 1762) believed dementia is a mental illness. Overall, 23% (*n* = 1362) of respondents selected ‘true’ or ‘I don’t know’ when asked if dementia was a part of the normal ageing process.

**Fig. 3:**
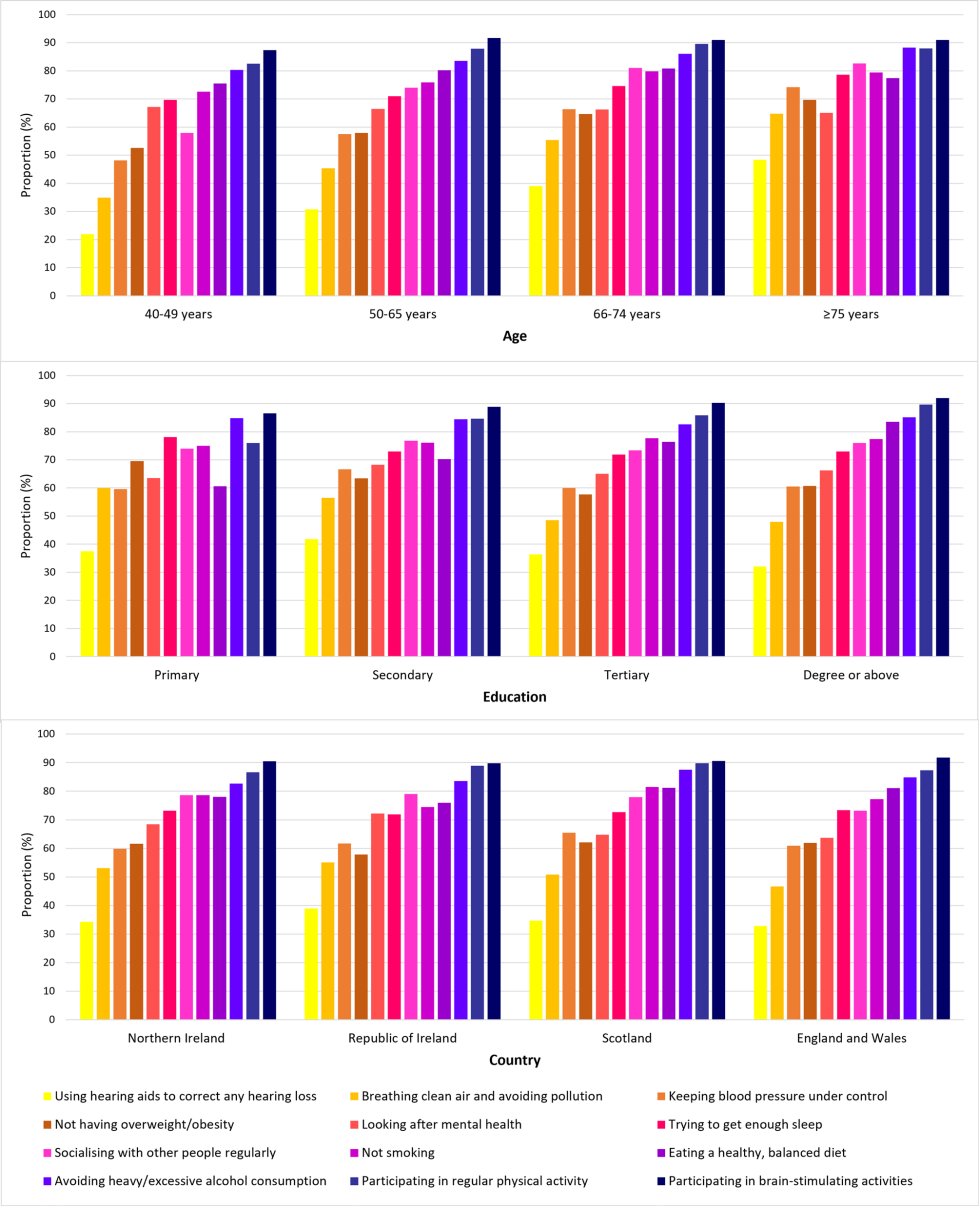
Awareness of modifiable factors by age, education level and country

Most respondents were aware that engaging in brain-stimulating activities (91%, *n* = 5542), regular exercise (88%, *n* = 5348) and avoiding heavy alcohol consumption (85%, *n* = 5148) is important for brain health. Awareness of eating a healthy balanced diet (80%, *n* = 4847), not smoking (77%, *n* = 4699), regular socialising (76%, *n* = 4602), getting sufficient sleep (73%, *n* = 4438), and looking after mental health (66%, *n* = 4036) was moderate. Factors recognised to a lesser extent, included the use of hearing aids to correct hearing loss (35%; *n* = 2106), avoiding pollution (50%; *n* = 3032), not having overweight/obesity (61%; *n* = 3708), and keeping blood pressure under control (62%; *n* = 3743). Differences in awareness between countries and age groups were observed for several modifiable factors. Overall, awareness was highest among the Republic of Ireland and older respondents (*≥* 66 years) and lowest among England/Wales and younger respondents (40–49 years). Awareness also differed by educational status; respondents with a lower educational level had poorer awareness of some (e.g., eating a healthy diet (*p* < 0.001), regular physical activity (*p* < 0.001)) modifiable factors. However, respondents educated to degree level or above had poorer awareness of other factors (e.g., having overweight/obesity (*p* < 0.01), and air pollution (*p* < 0.001)) compared to those educated to primary level or below. An overview of the associations between each modifiable factor and demographic categories is provided in Fig. 3, effect sizes (Cramer’s V) for which, ranged from 0.03 to 0.17.

### Engagement in risk and protective behaviours

Respondents’ engagement in risk and protective behaviours is provided in Table 2. To summarise, most of the sample were non-smokers (92%) and, nearly a third (29%) reported regular alcohol intake. In relation to potentially protective behaviours, most respondents believed that they ate a healthy diet (84%), engaged in regular exercise (65%), participated in brain-stimulating activities (67%), and socialised with others regularly (62%). However, participation in relaxing activities such as meditation, yoga, and mindfulness practice, was lower, as 18% of the sample engaged in such activities often.

There was moderate adherence to the MIND diet in the sample (*n* = 6410) with an average score of 9.01 (± 1.71) out of a possible 15 points. A breakdown of the frequency of consumption per MIND component is provided in Supplementary File 2 (Table S5). Notable dietary shortfalls included low intake of wholegrains (82% consumed fewer than three servings/day), green leafy vegetables (68% consumed fewer than six servings/week), and fish (46% consumed fewer than one serving/week). Full data on MIND diet component intake are provided in Supplementary Table S5. An overview of the total MIND score for categories of country, age, and education is provided in Table 3. Results indicated that respondents who lived in England and Wales, those aged 66–74 years and those educated to degree level or above, had higher MIND scores. From GP-PAQ responses (*n* = 6188), less than half of the sample (44.9%) were classed as active and more than half (55%) were inactive. Respondents categorised as inactive often had lower education (19.3%, primary level or below), were from NI (24.7%), were aged ≥ 75 years (15.6%), and/or were educated to primary level or below (19.3%).

The mean PSS-4 score among respondents was 4.89 (± 3.05) out of a possible 16. As outlined in Table 4, mean scores differed according to country, age, and education; those with higher PSS-4 scores were respondents who lived in NI, those aged 40–49 years, and those with tertiary level education.

### Facilitators for, and barriers to, changing behaviour to promote brain health

The facilitators selected most frequently by respondents were noticing problems with brain health (70%, *n* = 4235), receiving personal specific advice about what action to take (51%, *n* = 3068) and receiving a diagnosis of memory impairment or dementia (40%, *n* = 2391). Significant differences in the selection of facilitators between age, education, and country categories were identified. An overview of the associations between individual facilitators and demographic categories is provided in Supplementary File 2 (Table S6–S8). Some key findings were that respondents aged 40–49 years and those with higher educational attainment were more likely to select ‘if changes were fun and enjoyable’ compared to other age and education groups. In contrast, those with lower educational attainment were more likely to select ‘if changes were affordable’. Respondents aged ≥ 75 years had the greatest preference for ‘receiving personal specific advice about what to do’.

Barriers ranked highly included: the requirement to engage in unenjoyable activities (44%, *n* = 2639), lack of motivation (33%, *n* = 1965), lack of information about what to do (27%, *n* = 1626) and uncertainty that implementation of changes would help (27%, *n* = 1611). Facilitators also differed between demographic categories. For example, respondents aged 40–49 years were most likely to select the barriers ‘a lack of time’ and ‘a lack of motivation’ compared to other age groups. Respondents aged 66–74 years, respondents living in England/Wales and those educated to degree level or above, identified with ‘If I had to start doing activities that I do not enjoy’ more frequently compared to other age, country, and education groups. In contrast, respondents with lower educational attainment selected ‘If making changes were expensive’ and ‘A lack of information about what to do’, to a greater extent compared to those with higher educational attainment. An overview of the associations between individual barriers and demographic categories is provided in Supplementary File 2 (Tables S6–S8).

## Discussion

This study collected cross-sectional data on behaviours and awareness surrounding brain health, and the facilitators and barriers to future behaviour change, among ≥ 6800 respondents aged ≥ 40 years across the UK and Ireland. Although the sample was majority female (79%), white (99%), and highly educated, there was good representation across geographical regions and age groups.

Awareness of modifiable factors among the sample was mixed. Some protective factors (physical activity, healthy diet, and cognitive stimulation) were well-acknowledged, similar to previous research conducted both nationally and internationally [6, 16, 30, 31]. However, recognition of other risk factors (i.e., hearing loss, air pollution, hypertension, and obesity) was limited, similar to previous studies conducted among young Scottish adults [15] and Irish adults [19]. Global evidence has also suggested a limited understanding of the role of cardiometabolic risk factors (such as hypertension and obesity) in dementia risk reduction [6, 10, 31, 32] and reinforces a crucial need to improve awareness of these factors and provide individuals with the knowledge and tools to address them. Future work should also explore where individuals acquire this knowledge, to help guide the design of more targeted and effective public health messaging.

Awareness differed significantly between demographic groups for each modifiable factor. Generally, lower awareness was identified among younger adults and those with lower educational attainment, consistent with previous international research [30]. Future interventions aiming to improve awareness of dementia risk reduction, should therefore be tailored to the characteristics and needs of specific groups. Notably, these demographic differences also varied geographically, with the England/Wales group including a greater proportion of older and more highly educated respondents than the Republic of Ireland, which likely contributed to observed regional variation in awareness and behaviours. Beyond demographic composition, contextual factors such as national public health policy priorities, infrastructure, and exposure to dementia prevention messaging may also influence regional variation. These findings underscore the importance of designing brain health initiatives which are both demographically sensitive and contextually appropriate across regions. While many of the observed differences between groups were small in magnitude, effect sizes were calculated and included for key comparisons, and even modest differences can provide valuable insights for refining intervention design. At a population level, small shifts in awareness or behaviour across subgroups may translate into meaningful public health benefits. Future studies should consider adjusting for demographic variables to more clearly isolate country effects. Moreover, researchers should actively consider health equity during intervention design, to ensure that existing disparities, particularly among groups disproportionately affected by broader determinants of health, are not inadvertently reinforced [33, 34]. This includes addressing affordability-related barriers, which were more frequently reported among individuals with lower educational attainment in this sample. Such findings highlight the need for brain health initiatives and public health campaigns to consider socioeconomic constraints and reduce financial barriers, to support equitable access to brain health-promoting behaviours. Ultimately, these findings support co-designed approaches to brain health promotion. Whilst not analysed within this manuscript, free-text responses identifying potential actions to support brain health may offer valuable insights to inform future, contextually grounded intervention development.

Considering these findings, multi-level approaches which combines evidence-based subgroup- or individual-level strategies (e.g., health education) with low-agency population-level initiatives may be more effective in reaching diverse groups. Findings align with previous results from mass-media educational campaigns which aimed to raise awareness surrounding brain health in the Netherlands [35], Belgium [36] and Denmark [32]. Although one campaign identified an improvement in awareness among individuals with lower educational attainment [35], another [36], reported no change in awareness pre-post campaign among individuals with lower educational attainment. One solution to this, may be to run mass-media campaigns alongside smaller, targeted campaigns which have been co-produced with individuals from the target group and relevant stakeholders, such as public health bodies and media representatives [32, 34, 37]. These smaller campaigns could leverage non-traditional methods, such as dissemination of health information via messaging platforms (e.g., WhatsApp, Instagram) [38], and social media campaigns, which may include the use of social media influencers [38–40], alongside in-person group-based information sessions.

The provision of information (psychological capability) surrounding the promotion of brain health was also identified as a substantial barrier (lack of information) and a facilitator (provision of information) for behaviour change, similar to previous research [17, 36, 41]. Psychological capability refers to the mental processes required to engage in a behaviour, such as knowledge, comprehension, memory, and reasoning. In this context, a lack of psychological capability was reflected in respondents reporting limited awareness or understanding of what actions to take to support brain health. In relation to this, respondents also expressed uncertainty surrounding the benefits of behaviour change (beliefs about capability). Acknowledging the role of social influence as a facilitator, delivery of information from sources deemed trustworthy and legitimate by the target population, may help to improve both psychological capability and/or beliefs about capabilities. This could help to alleviate doubts about the credibility of information and in turn, potentially minimise sharing of misinformation [13, 42]. Consideration should also be given to the positive framing of information, using terms involving the promotion of brain health, and shifting away from focusing on the prevention of dementia [31, 43, 44].

Although psychological capability (which may be targeted via health education) was frequently reported as a barrier and facilitator for future behaviour change to promote brain health, behavioural drivers often act synergistically and should be considered in combination. Other prevalent facilitators were linked to social opportunity/social influences. Similarly, data from this study also suggested that both fear, and self-identity, were linked to brain health. For instance, a substantial proportion of respondents who self-identified as overweight still rated their general health positively, suggesting that perceived risk may not always align with the presence of modifiable factors. Combined, such findings align with previous research which suggest individuals are most likely to take preventive action when they believe they are susceptible to developing the condition and external cues are provided [17, 25, 41, 43, 45]. Noticing early cognitive changes was a more prevalent facilitator than receiving a formal diagnosis, suggesting that early subjective concerns may preserve a sense of agency, while diagnosis could, for some, trigger fatalism. This also suggests that for many, the cue to taking preventative action occurs at/or following the presentation of cognitive symptoms. This may be detrimental, as at this time point, it may be too late to prevent or delay dementia [44, 46]. Future work should therefore explore how best to provide appropriate cues to action early, to encourage the adoption of preventative behaviours. Although, this also highlights the potential for future campaigns to screen and identify individuals at-risk and to motivate their adoption of brain health promoting behaviours, in the pre-clinical/prodromal phase, prior to occurrence of tangible cognitive decline.

Other barriers to behaviour change identified were related to emotion (needing to engage in activities they did not enjoy) and optimism (lack of self-motivation). Yet a lack of self-motivation as a barrier to behaviour change was reported less frequently by respondents in this study compared to results from the Global Brain Health Survey (33% vs. 22%) [17]. Acknowledging the role of automatic motivation, this study contributes to evidence which emphasises the importance of using co-design principles within future behaviour change interventions, to nourish enjoyment, and improve efficacy [41].

Consumption of a healthy, balanced diet could contribute towards better brain health [47]. Regarding current behaviours, most respondents stated they had a healthy diet. The mean MIND score within this survey was higher compared to other study populations both in the UK [48], and internationally [26, 49, 50], which have generally ranged between 6 and 7 out of 15. This may be due to both the use of convenience sampling within this study (resulting in a highly educated, white and majority female population), and the use of a dietary screener, rather than a more granular dietary assessment tool (e.g., FFQ or 24-hr dietary recall). A previous qualitative study among Northern Irish adults which explored the facilitators and barriers to the MIND diet, did however, report similarly low intakes of wholegrains (44% less than twice p/w), fish (72% less than twice p/w), and beans/legumes (76% less than twice per week). Such components (e.g., fish and wholegrains) are often found in diets associated with potentially neuroprotective effects (e.g., MIND diet, Mediterranean diet). In addition to dietary behaviour, the BHLS also captured participants’ beliefs about what constitutes a healthy diet, which may inform future research on dietary knowledge and its relationship with brain health behaviours. Further investigation is needed to understand the barriers and facilitators to increasing consumption of specific food components, particularly among UK and Irish populations, to better support brain health.

Over half of all respondents stated they engaged in regular exercise, but only 27% were categorised as active according to GP-PAQ. However, categorisation for the GP-PAQ derives from time spent engaging in both structured physical exercise and occupational exercise [28]. Thus, a potential explanation for such findings may involve the high proportion of retired respondents in this sample. Although, additional analyses were performed to stratify the sample and remove respondents with no occupational activity level (e.g., retired, unemployed, other). Results showed the percentage of respondents deemed physically active according to GP-PAQ remained relatively low, at 35%. Ultimately, this reflects previous research which has suggested many UK and Irish adults fail to meet national recommended physical activity guidelines [51, 52]. It may also highlight a reduction in social desirability/conformity bias when physical activity is assessed using a formal assessment tool, versus a simple question which asks about perceived physical activity levels. A third of the sample reported regular consumption of alcohol and a further 5% suggested their consumption was excessive. Incorporating the link between heavy alcohol consumption and dementia risk within future public health campaigns may increase perceived susceptibility among the public, which was acknowledged as a facilitator for lifestyle change [53–55].

This study has several strengths. The design of the BHLS was evidence-based, developed with PPI representatives, and underpinned by behaviour change frameworks. Data was captured to explore awareness, beliefs, and behaviours surrounding brain health, across a large sample size. However, of the 9,127 individuals who accessed the survey, 2,226 did not complete consent and were excluded (24% attrition). This level of drop-out was expected due to absence of incentives. A key limitation was the use of convenience sampling which led to a sample predominantly composed of white, female, and well-educated respondents. This also resulted in variations in demographic characteristics (e.g., age, gender, and educational attainment) across countries, meaning observed differences may reflect demographic composition rather than true country effects. Future research should adjust for these factors to better isolate country-specific influences, as well as to improve sample diversity to enhance the generalisability of findings to the wider UK and Irish population. Secondly, the BHLS was completed online, which may have limited access by those living in data poverty and/or those with poor digital literacy. Future research may consider re-administration of the BHLS using both an online and paper format, to under-represented groups to improve representation and comparability. Additionally, awareness was assessed using recognition-based items, a common approach in dementia literacy research. However, this method cannot confirm whether responses reflected true knowledge. Future research could incorporate open-ended items to assess awareness more robustly. In addition, the order in which survey questions were presented, with questions assessing behaviour, preceding those which examined awareness, may have introduced bias, although this was intended to reduce priming and reflect natural response flow. Data collection for this study also occurred during the global COVID-19 pandemic, which may have influenced participants’ responses and behaviours. This context should be considered when interpreting study results. Additionally, sub-group comparisons were not adjusted for potential confounding variables. As such, observed differences between countries or demographic groups may reflect underlying disparities in age, education, or other characteristics, rather than true effects. Future research should employ multivariable analytical approaches to more accurately isolate the influence of specific factors.

## Conclusion

This study explored public awareness, beliefs, and behaviours surrounding brain health using a sample of 6816 adults aged ≥ 40, living across the UK and Ireland. Findings have highlighted the need for a swift and effective pathway for translation of scientific evidence surrounding brain health, into accessible, practical, and trustworthy information for the public. Awareness, beliefs, and behaviours surrounding brain health differed between demographic groups. Therefore, future interventions and policies should acknowledge the impact of these factors and aim to reduce existing population inequalities in this field and not indirectly exacerbate them. Combining, co-designed, evidence-based subgroup- or individual-level approaches (such as health education), alongside low-agency population-level approaches may help to facilitate this.

**Table 1:** Overview of respondent demographic characteristics

Demographic	Category	%	*n* =
Country	NI	10.5	718
	Republic of Ireland	25.3	1722
	Scotland	11.7	796
	England	50.3	3429
	Wales	2.2	151
Age (years)	40–49	10.2	696
	50–65	46.5	3168
	66–74	31.5	2144
	75+	11.9	808
Gender	Male	21.3	1438
	Female	78.5	5299
Education	Primary education	1.9	126
	Secondary education	18.0	1214
	Tertiary education	15.5	1045
	Degree level and above education	63.7	4295
Ethnicity	White	98.5	6650
	Non-White	1.2	80
	Prefer not to answer	0.4	25
Weight status	Underweight	1.5	102
	About the right weight	39.1	2621
	A bit overweight	37.2	2490
	Overweight	16.7	1115
	A lot overweight	5.50	368
Self-rated overall health	Excellent	12.2	818
	Very Good	39.7	2662
	Good or above	33.1	2223
	Fair	12.5	841
	Poor	2.3	152
	I don’t know	0.2	11
Self-rated brain health	Excellent	3.6	420
	Very Good	30.8	2060
	Good or above	41.9	2804
	Fair	15.7	1048
	Poor	1.9	130
	I don’t know	3.4	228

**Table 2:** Distribution of responses for engagement in seven behaviours relating to brain health

Potential responses	Listed behaviour						
	Eating a healthy diet *n* (%)	Exercising regularly *n* (%)	Participating in relaxing activities *n* (%)	Participate in brain-stimulating activities *n* (%)	Socialise with others *n* (%)	Drink alcohol *n* (%)	Smoking *n* (%)
Never	7 (0.1)	175 (2.6)	1809 (27.2)	100 (1.5)	38 (0.6)	905 (13.6)	6120 (92.1)
Rarely	83 (1.2)	663 (10.0)	1865 (28.1)	402 (6.1)	459 (6.9)	1512 (22.8)	127 (1.9)
Sometimes	1002 (15.1)	1500 (22.6)	1803 (27.1)	1675 (25.2)	2054 (30.9)	2277 (34.4)	117 (1.8)
Often	3312 (49.9)	2123 (31.9)	834 (12.6)	2361 (35.5)	3012 (45.3)	1595 (24.0)	184 (2.8)
Very often	2239 (33.7)	2184 (32.9)	333 (5.0)	2105 (31.7)	1090 (16.3)	356 (5.4)	97 (1.5)

**Table 3:** Mean MIND scores by country, age, and educational level

Demographic variable	Categories	MIND score (Mean ± SD)	*n* =	One-way ANOVA *p*-value =	η² (eta-squared)
Country of residence	NI	8.86 ± 1.72	672	<0.001	0.018
	Republic of Ireland	8.79 ± 1.71	1555		
	Scotland	8.90 ± 1.68	756		
	England & Wales	9.16 ± 1.90	3427		
Age (years)	40–49	8.75 ± 1.82	640	<0.001	0.009
	50–65	9.02 ± 1.72	2968		
	66–74	9.10 ± 1.67	2041		
	75+	8.90 ± 1.61	761		
Education level	Primary education or below	8.44 ± 1.80	119	<0.001	0.004
	Secondary education	8.61 ± 1.72	1124		
	Tertiary education	8.89 ± 1.70	995		
	Degree level or above	9.17 ± 1.68	4106		

**Table 4:** Mean PSS-4 scores by country, age, and education level

Demographic variable	Categories	Mean PSS-4 score (± SD)	*n* =	One-way ANOVA *p*-value =	η² (eta-squared)
Country of residence	NI	5.12 ± 3.02	628	0.009	0.036
	Republic of Ireland	5.03 ± 3.04	1374		
	Scotland	4.95 ± 3.05	700		
	England & Wales	4.77 ± 3.05	3266		
Age (years)	40–49	6.21 ± 3.13	599	< 0.001	0.003
	50–65	5.13 ± 3.07	2756		
	66–74	4.34 ± 2.93	1914		
	75+	4.32 ± 2.73	713		
Education level	Primary education or below	4.76 ± 3.08	100	< 0.001	0.002
	Secondary education	5.11 ± 3.13	1003		
	Tertiary education	5.12 ± 3.12	916		
	Degree level or above	4.76 ± 3.00	3899		

## Supplementary Information

Below is the link to the electronic supplementary material.

Supplementary Material 1

Supplementary Material 2

## Data Availability

The data that support the findings of this study are available from the corresponding author upon reasonable request.
