# Supplementary material for: Awareness, facilitators, barriers, and behaviours surrounding brain health: a large-scale cross-sectional survey of adults across UK and Ireland
Source: BMC Public Health. 2025 Oct 1;25:3279. doi: 10.1186/s12889-025-24175-0 (PMC12487460; doi:10.1186/s12889-025-24175-0)
Supplement: Supplementary file 1 — Supplementary Material 1 [file 12889_2025_24175_MOESM1_ESM.docx]

**Title of Project: Public opinions and lifestyle practices relating to brain health: A survey of adults living across the UK and Ireland.**

[Participant information sheet]

[Participant consent form]

**This survey aims to understand your opinions on brain health and the factors that you think may influence this.**

**First, we would like to check that you are eligible to participate in the survey. Please confirm your age and where you live**

**S1. How old are you?**(Please select one)

- 16-39 years old [EXCLUDED]
- 40-49 years old
- 50-65 years old
- 66-74 years old
- 75+ years old

**S2. Where do you currently live?**(Please select one)

- Northern Ireland
- Ireland
- Scotland
- England
- Wales
- I live outside the UK or the Republic of Ireland [EXCLUDED].

__________________________________________________________________________________

If individual selects appropriate answers to reflect they are eligible, the following message will be displayed: **Thank you. The answers you have provided so far tells us you are eligible to participate in this survey.**
_________________________________________________________________________

**Section 1. This section is about your personal information, followed by thoughts about your own general health and brain health, including any medical conditions you may have.**

- 1. **What is your gender?**(Please select one)
- Male
- Female
- Non-binary
- I’d prefer not to answer this question
  1. **What is your ethnicity?**(Please select one)
- White
- Chinese
- Irish Traveller
- Indian
- Pakistani
- Black Caribbean
- Black African
- Black Other
- Mixed Ethnic Group
- Other Ethnic Group
- I’d prefer not to answer this question
  1. **What is your current employment status?**(Please select one answer)
- Full-time employee
- Part-time employee
- Self-employed / Freelance
- Unemployed
- Retired
- Other (Please specify)
- I’d prefer not to answer this question
  1. **What is your highest level of education?**(Please select one answer)
- Primary Education or below
- Secondary Education (GCSE/GCE levels/ Intermediate / Junior/ Group Certificate or equivalent)
- Tertiary Education (Diploma/ A levels/ Leaving Certification or equivalent)
- Degree Level Education (Degree level or equivalent e.g. NVQ Level 4, Higher National Diploma or Certificate)
- Above Degree Level Education (Postgraduate degree/diploma or higher (PhD)
- I’d prefer not to answer this question

**First, we would like to ask you about your overall health.**

- 1. **How would you rate your overall health?**(Please select one)
- Excellent
- Very good
- Good
- Fair
- Poor
- I don’t know
  1. **Do you have any of the following chronic conditions or illnesses?**(Please select one)
- I have no chronic conditions or illnesses
- Angina
- Heart Disease
- Any Circulatory Disease (E.g. Peripheral Arterial Disease/Peripheral Vascular Disease)
- Stroke/Transient Ischaemic Attack
- Diabetes
- High Blood Pressure
- High Cholesterol
- Dementia
- Alzheimer’s Disease
- Mild Cognitive Impairment
- Depression
- Anxiety
- Cancer
- Chronic Obstructive Pulmonary Disease
- Chronic Kidney Disease
- Asthma
- Arthritis
- Chronic Back Pain
- Other chronic illness/condition(s) not listed above [Please specify in free text box]
- I’d prefer not to answer this question
- I am not sure [Please specify in free text box]
  1. **Which of the following best describes how you view your weight relative to your height at the moment?**
     (Please select one)
- Underweight
- About the right weight
- A bit overweight
- Overweight
- A lot overweight

**Next we would like to ask you about your brain health.**

**Brain health involves the brain’s capability to remember, comprehend and learn information, and the ability to think strategically to make everyday decisions, and this impacts a person’s ability to function well in daily life and work.**

- 1. **How often do you think about your own brain health?**(Please select one)
- Never
- Rarely
- Sometimes
- Often
- Very often
  1. **How would you rate your brain health?**(Please select one)
- Excellent
- Very good
- Good
- Fair
- Poor
- I don’t know

**Section 2. This section is about your current lifestyle behaviours**

**The information you provide will help us to understand more about how and why the general adult population engages in certain behaviours.**
**Please try to be as accurate and as honest as possible with your answers, selecting the option that you feel most closely matches your typical habits.**

- 1. **Firstly, have you taken any steps with your own lifestyle, specifically to protect your brain health in the future?**(Please select one)
- Yes
- No
- I don’t know

**2.2 Below is a list of lifestyle behaviours.
How often do you engage in each lifestyle behaviour?**(Please select one answer in response to each statement)

|  | **Behaviour** | **Very often** | **Often** | **Sometimes** | **Rarely** | **Never** |
| --- | --- | --- | --- | --- | --- | --- |
| 2.2.1 | How often do you eat a healthy diet? |  |  |  |  |  |
| 2.2.2 | How often do you engage in regular exercise (e.g. 4-5 times per week)? |  |  |  |  |  |
| 2.2.3 | How often do you participate in relaxing activities (e.g. meditation, yoga, mindfulness)? |  |  |  |  |  |
| 2.2.4 | How often do you drink alcohol? |  |  |  |  |  |
| 2.2.5 | How often do you smoke? |  |  |  |  |  |
| 2.2.6 | How often do you participate in activities that stimulate your brain (e.g. crosswords, learning new things)? |  |  |  |  |  |
| 2.2.7 | How often do you socialise with other people (e.g. spending time with family and friends) |  |  |  |  |  |

**2.3 Now we would like to ask you a few questions about your dietary intake. The information you provide will help us to understand more about food consumption among the general adult population.**
 
**Please try to be as accurate and as honest as possible with your answers and select the serving amount that you feel most closely matches your typical eating habits.**
**If you're unsure, examples of what one serving of each food might look like are featured below each question.**

| *Question* | *Example of serving* | *Answer choices* |
| --- | --- | --- |
| **2.3.1** How many servings of whole-grains do you eat on average in a **typical DAY?**  (This includes porridge oats, muesli, wholegrain, oat or wheat-based breakfast cereal, multi-grain, wholemeal or rye bread, brown rice, bulgur wheat, wholegrain pasta, Ryvitas or oatcakes) | 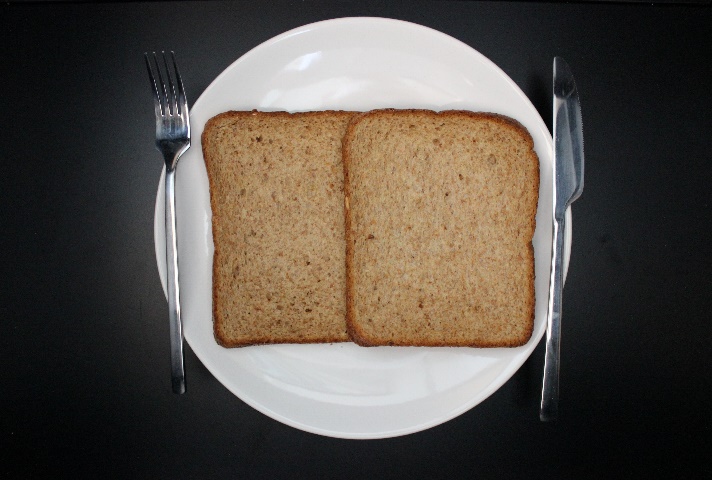*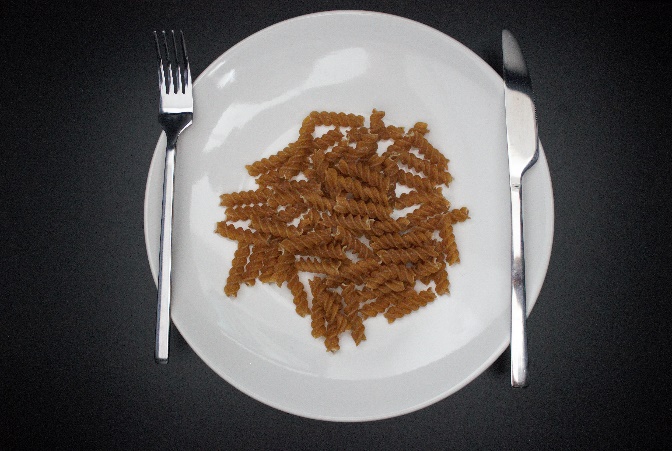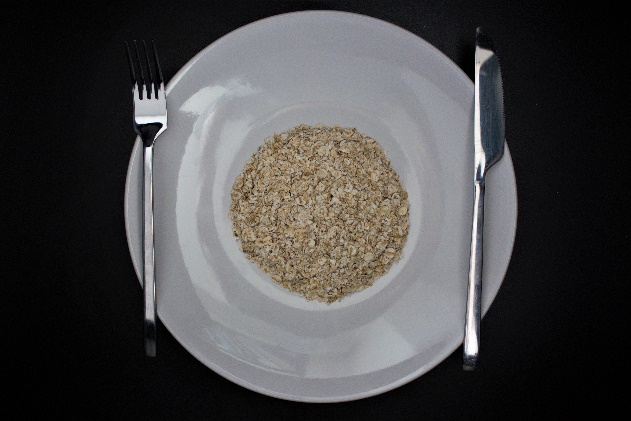*1 serving may look like = | 0 serving  1 serving  2 servings  3 servings  4 servings  5 servings  6 servings  7 servings or more |
| **2.3.2** How many servings of butter or margarine do you have in a **typical DAY?** 1 serving may look like = | 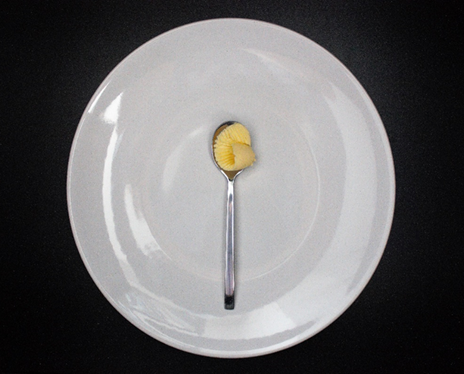 | 0 serving  1 serving  2 servings  3 servings  4 servings  5 servings  6 servings  7 servings or more |
| **2.3.3** How many servings of green leafy vegetables do you eat in **a typical WEEK**?  (This includes raw salad greens, spinach, cabbage, broccoli, kale, chard or collard)  1 serving may look like = | 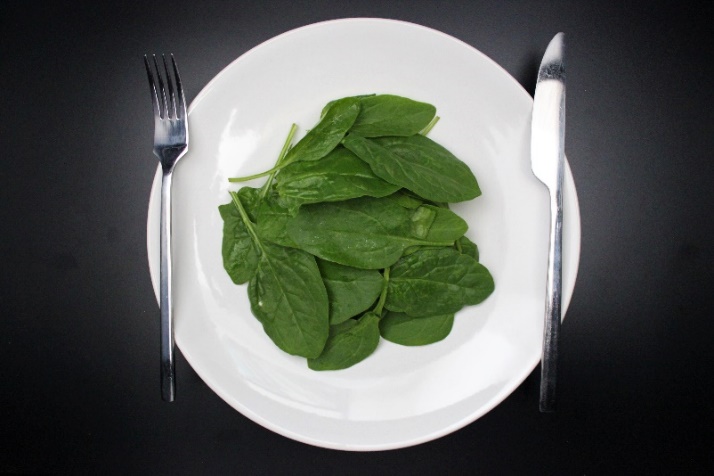  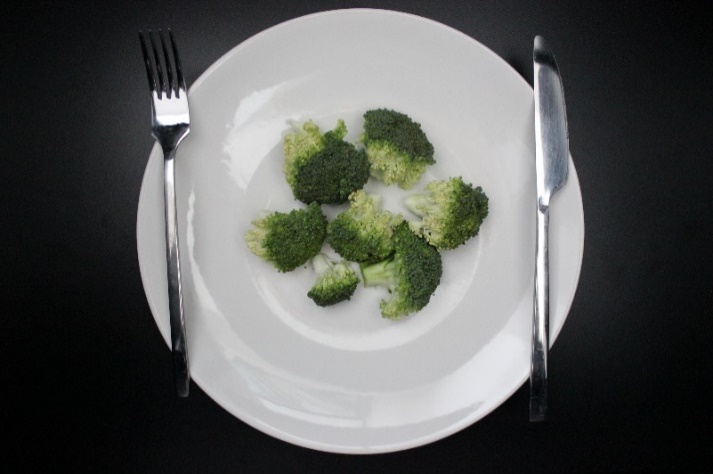 | Less than 1 serving per week  1 serving  2 servings  3 servings  4 servings  5 servings  6 servings  7 servings or more |
| **2.3.4** How many servings of vegetables (excluding green leafy vegetables) do you eat in a **typical WEEK**?    (This includes vegetables such as carrots, peppers, tomatoes, mushrooms, onions)  1 serving may look like = | 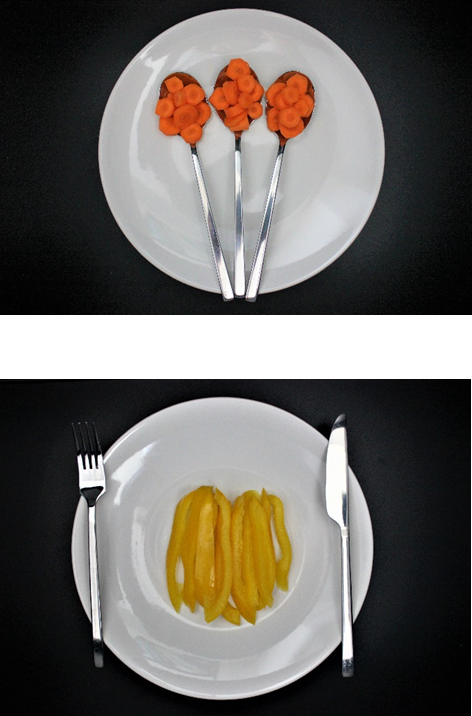 | Less than 1 serving per week  1 serving  2 servings  3 servings  4 servings  5 servings  6 servings  7 servings or more |
| **2.3.5** How many servings of red or processed meat do you eat in a **typical WEEK?**    (This includes pork, beef, lamb, bacon, ham, sausages, meat pies or other meat products)  1 serving may look like = | 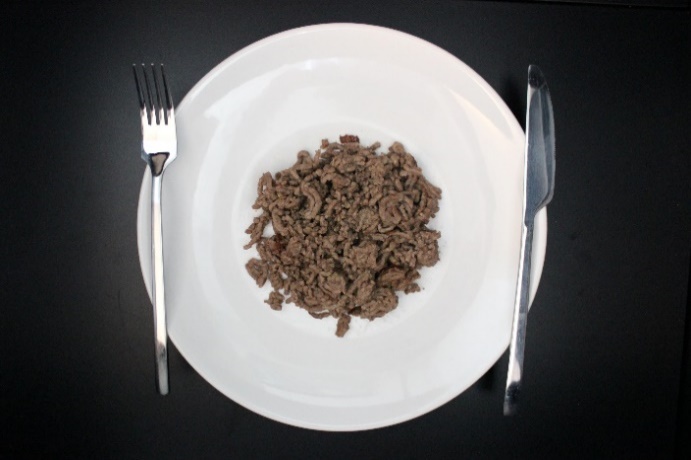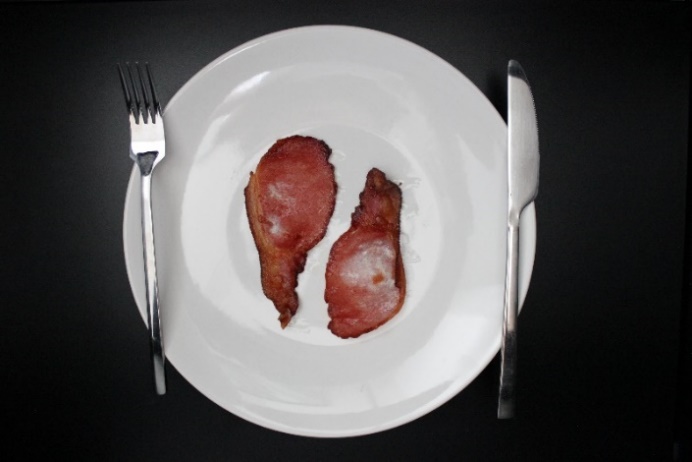 | 0 serving  1 serving  2 servings  3 servings  4 servings  5 servings  6 servings  7 servings or more |
| **2.3.6** How many servings of fish or shellfish do you eat in a **typical WEEK?**    (This includes tuna, cod, haddock, salmon, mackerel, herring, sardines and any tinned varieties or shellfish. Excludes crumbed, breaded or battered fish)  1 serving may look like = | 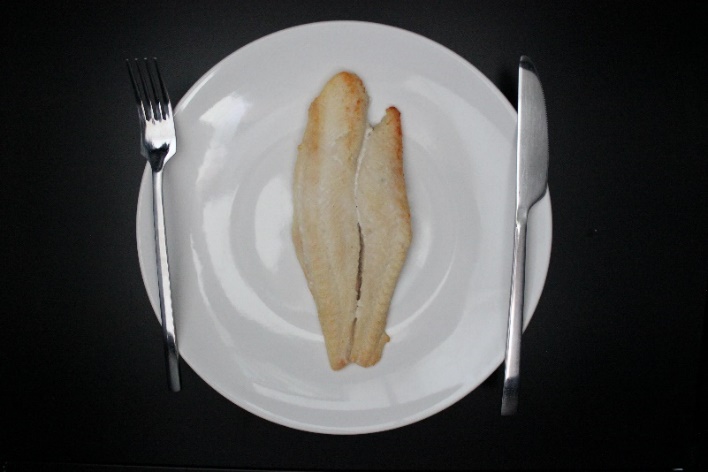 | Less than 1 serving per week  1 serving  2 servings  3 servings  4 servings  5 servings  6 servings  7 servings or more |
| **2.3.7** How many servings of **chicken or turkey (non-fried)** do you eat in a **typical WEEK**?  1 serving may look like = | 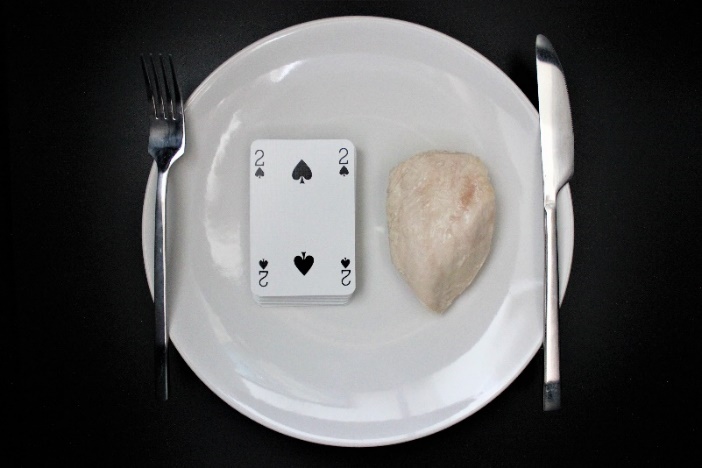 | Less than 1 serving per week  1 serving  2 servings  3 servings  4 servings  5 servings  6 servings  7 servings or more |
| **2.3.8** How many servings of beans and pulses do you eat in **a typical WEEK**?  This includes beans (green beans, broad beans, kidney beans), peas (garden peas, chickpeas) and lentils (green, red and yellow)  1 serving may look like = | 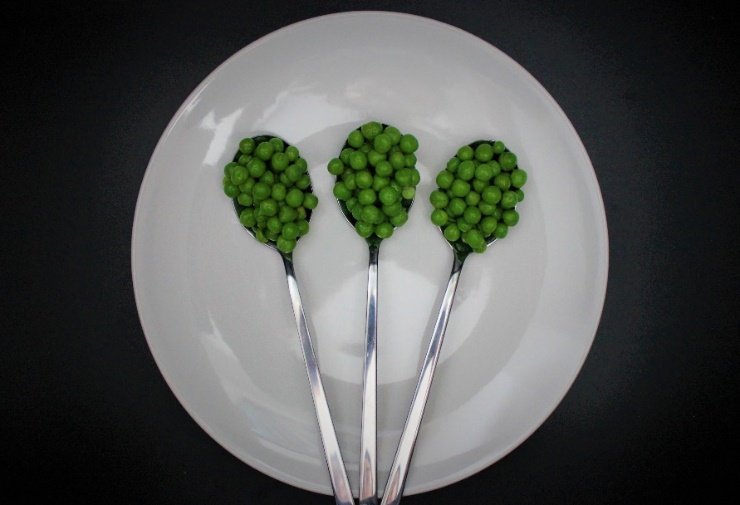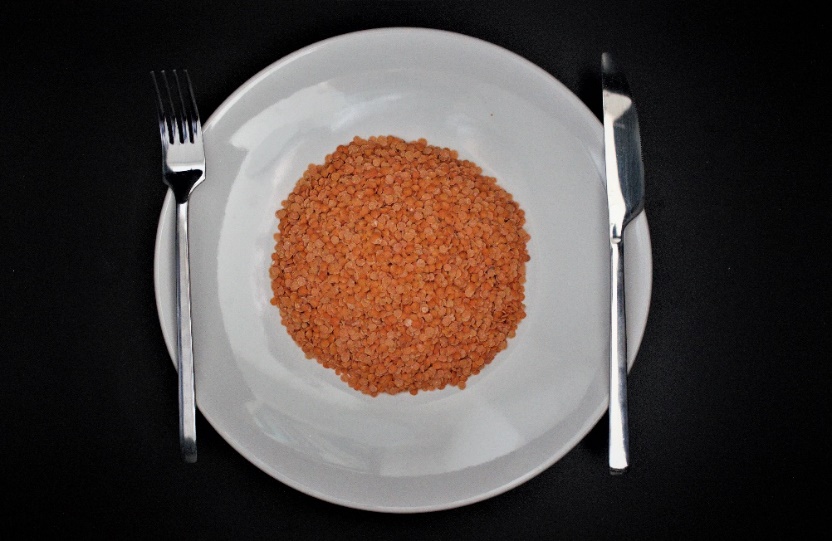 | Less than 1 serving per week  1 serving  2 servings  3 servings  4 servings  5 servings  6 servings  7 servings or more |
| **2.3.9** How many servings of cheese do you eat in a **typical WEEK**?    (This includes cheddar cheese, cream cheese, ricotta, cottage cheese or any other cheese).  1 serving may look like = | 1  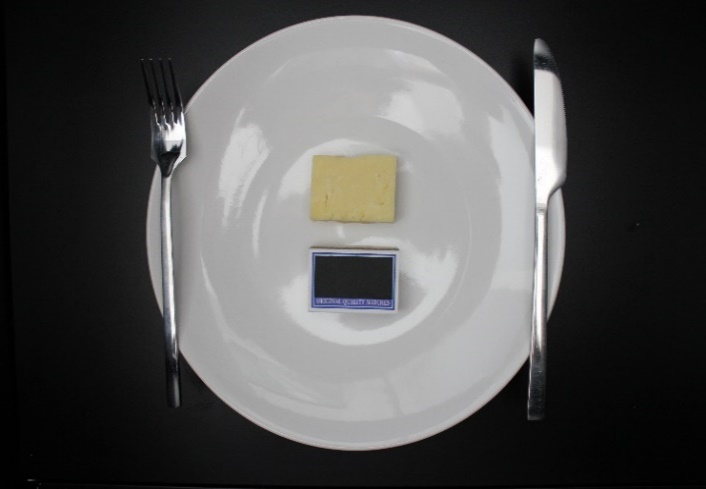 | Less than 1 serving per week  1 serving  2 servings  3 servings  4 servings  5 servings  6 servings  7 servings or more |
| **2.3.10** How many servings of natural/raw nuts do you eat in **a typical WEEK?**    (This does NOT include salted or coated nuts)  1 serving may look like = | 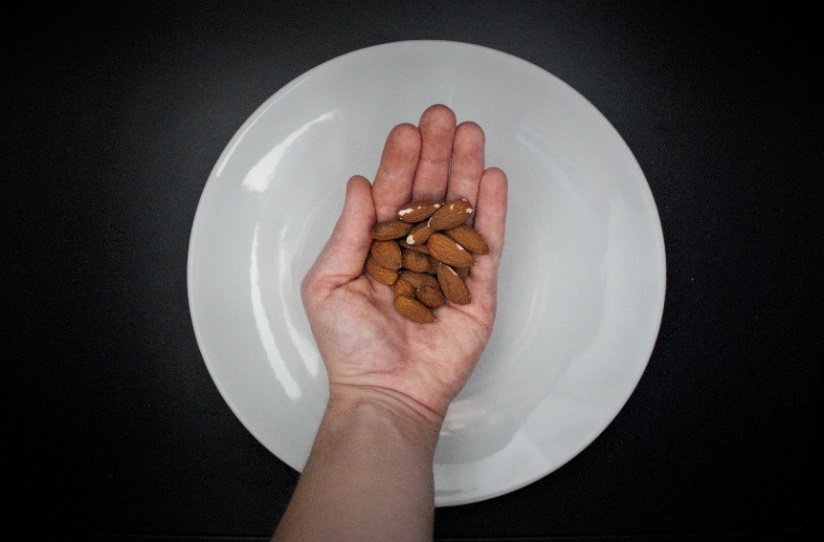 | Less than 1 serving per week  1 serving  2 servings  3 servings  4 servings  5 servings  6 servings  7 servings or more |
| **2.3.11** How many servings of berries do you eat in a **typical WEEK**?  (This includes strawberries, blueberries, blackberries, raspberries or other berries)  1 serving may look like = | 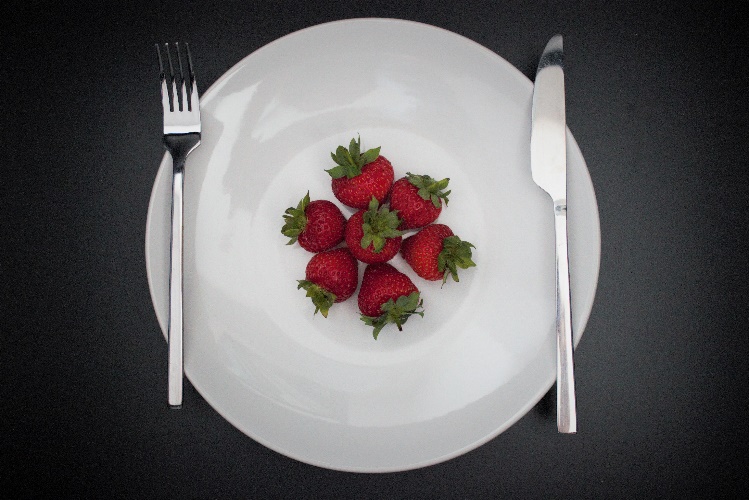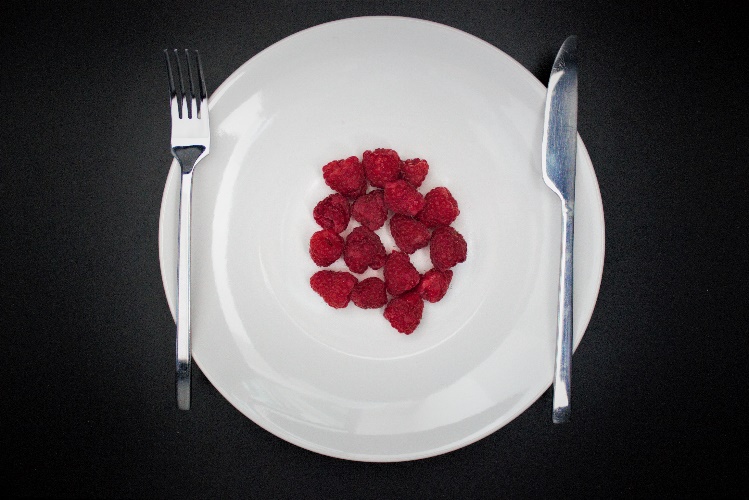 | Less than 1 serving per week  1 serving  2 servings  3 servings  4 servings  5 servings  6 servings  7 servings or more |
| **2.3.12** How many glasses of wine do you drink in a **typical WEEK?**    1 glass = 1 small glass (125ml)  1 serving may look like = | 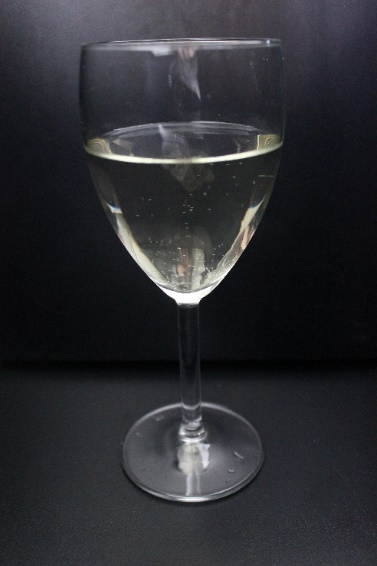 | I do not drink alcohol  Less than 1 glass per week  1 glass  2 glasses  3 glasses  4 glasses  5 glasses  6 glasses  7 glasses or more |
| **2.3.13** How many times do you consume dessert/sweet foods in a **typical WEEK?**  (Includes biscuits, buns, pastries, chocolate, sweets, other desserts and sweet carbonated beverages)  1 serving may look like = | 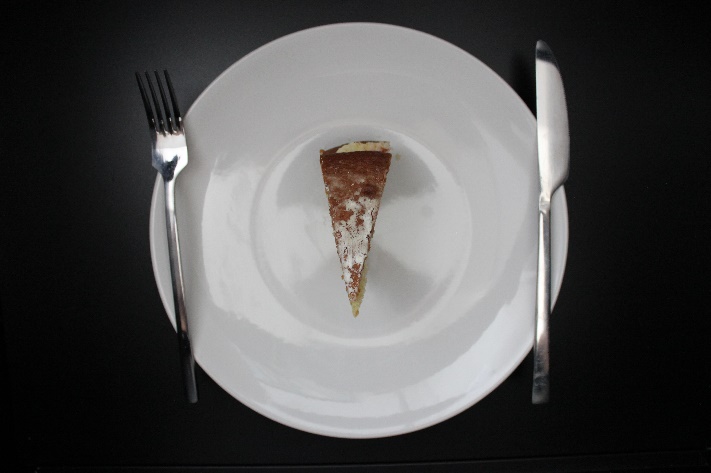  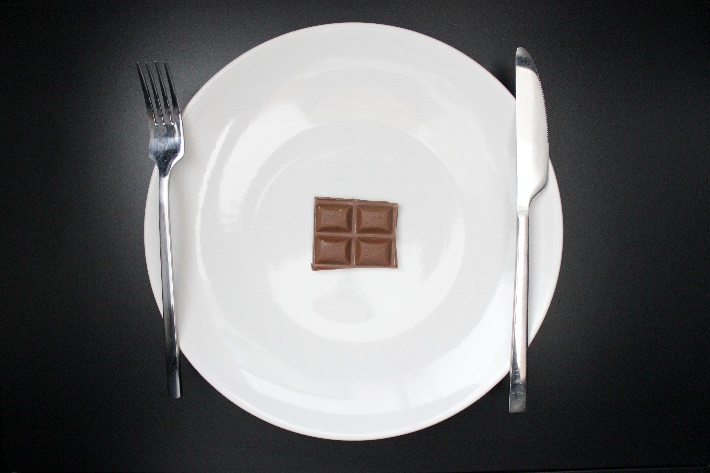 | Less than 1 serving per week  1 serving  2 servings  3 servings  4 servings  5 servings  6 servings  7 servings or more |
| **2.3.14** How many times do you consume take-away food in a **typical WEEK?** | 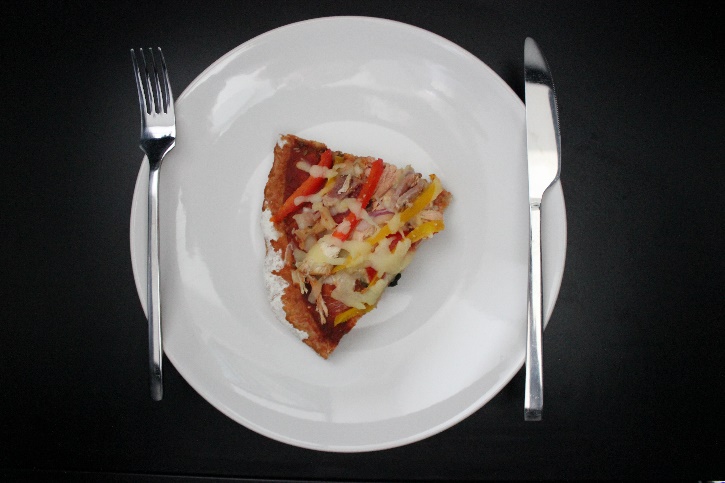 | Less than once per week  Once per week  Twice per week  3 times per week  4 times per week  5 times per week  6 times per week  7 times per week or more |
| Which **oil/fat** do you use the most **for cooking**? |  | Olive oil  Vegetable oil  Butter/Margarine  Coconut oil  Lard or any type of animal fat  Sunflower oil  Low-calorie spray oil  Other [*Please specify using free text]* |

**_________________________________________________________________________________**

**Section 3. This section is about your beliefs towards eating a healthy diet

Now we know more about your diet, we would like to better understand your beliefs about eating a healthy diet.
Please tell us to what extent you agree or disagree with each statement below. There are no right or wrong answers, simply read each statement and answer it according to how you think and feel.**

**To what extent do you agree or disagree with the following statements:**

| **Statement** | **Strongly  Disagree** | **Disagree** | **Neither agree nor**  **disagree** | **Agree** | **Strongly Agree** |
| --- | --- | --- | --- | --- | --- |
| **3.1.1 I** know what a healthy diet consists of  (Please select one answer in response to the statement) |  |  |  |  |  |
| **3.1.2** I am confident that I can eat a healthy diet  (Please select one answer in response to the statement) |  |  |  |  |  |
| **3.1.3** My alcohol consumption often negatively impacts the likelihood that I will make healthy food choices  (Please select one answer in response to the statement) |  |  |  |  |  |
| **3.1.4** Eating a healthy diet is difficult during holiday periods  (Please select one answer in response to the statement) |  |  |  |  |  |
| **3.1.5** I regularly try to make healthy food choices  (Please select one answer in response to the statement) |  |  |  |  |  |
| **3.1.6** I am confident in my ability to cook healthy meals  (Please select one answer in response to the statement) |  |  |  |  |  |
| **3.1.7 I** often struggle to know how to cook healthy meals  (Please select one answer in response to the statement) |  |  |  |  |  |
| **3.1.8 It** is difficult to eat a healthy diet during social events with my family/friends  (Please select one answer in response to the statement) |  |  |  |  |  |
| **3.1.9** My busy lifestyle means it is difficult to prepare/cook healthy meals   (Please select one answer in response to the statement) |  |  |  |  |  |
| **3.1.10** My workplace environment makes it difficult to eat a healthy diet  (Please select one answer in response to the statement) |  |  |  |  |  |
| **3.1.11** It is expensive to eat a healthy diet  (Please select one answer in response to the statement) |  |  |  |  |  |
| **3.1.12** My family responsibilities mean it is difficult for me to eat a healthy diet  (Please select one answer in response to the statement) |  |  |  |  |  |
| **3.1.13** I often dislike the taste of healthy foods  (Please select one answer in response to the statement) |  |  |  |  |  |
| **3.1.14** I regularly prepare and plan my meals ahead of time  (Please select one answer in response to the statement) |  |  |  |  |  |
| **3.1.15 I** enjoy trying and cooking new foods  (Please select one answer in response to the statement) |  |  |  |  |  |
| **3.1.16** I believe that eating a healthy diet could help me maintain or improve my overall health and wellbeing  (Please select one answer in response to the statement) |  |  |  |  |  |

**________________________________________________________________________**

**Section 4. In the next few questions we would like to ask you a few questions about your physical activity levels**

- 1. **Please tell us the type and amount of physical activity involved in your work:**

(Please select one answer)

- I am not in employment (e.g. retired, retired for health reasons, unemployed, full-time carer etc.)
- I spend most of my time at work sitting (such as in an office)
- I spend most of my time at work standing or walking. However my work does not require much intensive physical effort (e.g. shop assistant, hairdresser, security guard, childminder, etc.)
- My work involves definite physical effort including handling of heavy objects and use of tools (e.g. plumber, electrician, carpenter, cleaner, hospital nurse, gardener, postal delivery workers etc.)
- My work involves vigorous physical activity including handling of very heavy objects (e.g. scaffolder, construction worker, refuse collector, etc.)

**4.2 During the last week, how many hours did you spend on each of the following activities?**

| Activities | None | Some, but less than 1 hour | 1 hour, but less than 3 hours | 3 hours or more |
| --- | --- | --- | --- | --- |
| - - 1. Any form of planned physical exercise (such as swimming, jogging, aerobics, football, tennis, gym workout etc.)   (Please select one answer in response to the statement) |  |  |  |  |
| - - 1. Cycling (including cycling to work and during leisure)   (Please select one answer in response to the statement) |  |  |  |  |
| - - 1. Walking (including walking to work, shopping, for pleasure etc.)   (Please select one answer in response to the statement) |  |  |  |  |
| - - 1. Housework/Childcare   (Please select one answer in response to the statement) |  |  |  |  |
| - - 1. Gardening/DIY   (Please select one answer in response to the statement) |  |  |  |  |

- 1. **How would you describe your usual walking pace?**

(Please select one)

- Slow pace (i.e. less than 3mph)
- Steady average pace
- Brisk pace
- Fast pace (i.e. over 4 mph)

**_________________________________________________________________________

Section 5. In this section, we would like to know your thoughts about brain health.**

**Brain health involves the brain’s capability to remember, comprehend and learn information, and the ability to think strategically to make everyday decisions, and this impacts a person’s ability to function well in daily life and work.**

- 1. **There are several things that people can do (or avoid doing) to help protect their brain health in the future. Can you name any?**[Free text entry answer]
  2. **In your opinion, at what stage(s) in life should someone take action to look after their brain health?​**

(Please select all answers that apply)

- Childhood (birth to 12 years)
- Adolescence (13-18 years)
- Young adulthood (19-45 years)
- Middle age (46-65 years)
- Old age (>65 years)
- All of the above
  1. **Now we would like to better understand your beliefs about brain health.
     We would like to show you some statements and ask you to tell us to what extent you agree or disagree with each. There are no right or wrong answers here, simply read each statement and answer it according to how you think and feel.**

| **Statement** | **Strongly  Disagree** | **Disagree** | **Neither agree nor**  **disagree** | **Agree** | **Strongly Agree** |
| --- | --- | --- | --- | --- | --- |
| **5.3.1** I believe that I am likely to experience poor brain health in the future.  (Please select one answer in response to the statement) |  |  |  |  |  |
| **5.3.2** There is a strong possibility that my brain health will decline in the next 10 years.  (Please select one answer in response to the statement) |  |  |  |  |  |
| **5.3.3** The thought of my brain health declining scares me.  (Please select one answer in response to the statement) |  |  |  |  |  |
| **5.3.4** My feelings about myself would change if my brain health declined.  (Please select one answer in response to the statement) |  |  |  |  |  |
| **5.3.5** When I think about the possibility of my brain health declining, my heart beats faster.  (Please select one answer in response to the statement) |  |  |  |  |  |

- 1. **Below is a list of things which can help to maintain brain health. For each, please say how aware you were of its importance for brain health.**

| **Behaviour** | **I definitely knew this** | **I think I knew this** | **I did not know this, but it is unsurprising** | **I did not know this and I find it a little surprising** |
| --- | --- | --- | --- | --- |
| **5.4.1** Eating a healthy, balanced diet  (Please select one answer in response to the statement) |  |  |  |  |
| **5.4.2** Using hearing aids to correct any hearing loss  (Please select one answer in response to the statement) |  |  |  |  |
| **5.4.3** Keeping blood pressure under control  (Please select one answer in response to the statement) |  |  |  |  |
| **5.4.4** Participating in regular physical activity  (Please select one answer in response to the statement) |  |  |  |  |
| **5.4.5** Socialising with other people regularly (e.g. spending time with family and friends)  (Please select one answer in response to the statement) |  |  |  |  |
| **5.4.6** Participating in brain-stimulating activities (e.g. crosswords, learning new things)  (Please select one answer in response to the statement) |  |  |  |  |
| **5.4.7** Not smoking  (Please select one answer in response to the statement) |  |  |  |  |
| **5.4.8** Looking after mental health  (Please select one answer in response to the statement) |  |  |  |  |
| **5.4.9** Breathing clean air and avoiding pollution  (Please select one answer in response to the statement) |  |  |  |  |
| **5.4.10** Avoiding heavy/excessive alcohol consumption  (Please select one answer in response to the statement) |  |  |  |  |
| **5.4.11** Having overweight/obesity  (Please select one answer in response to the statement) |  |  |  |  |
| **5.4.12** Trying to get enough sleep  (Please select one answer in response to the statement) |  |  |  |  |

**Knowing that having a healthy lifestyle, including eating a well-balanced diet and participating in regular physical activity could maintain or improve your brain health, please answer the following questions.**

**5.5 What would motivate you the most to improve your lifestyle for your own brain health?**[SELECT **UP TO 3 OPTIONS** THAT YOU CONSIDER TO BE THE **MOST IMPORTANT**. PLEASE SELECT **AT LEAST ONE** OPTION].

1. If I noticed problems with my brain health (e.g. my memory worsened)
2. If I had been diagnosed with memory impairment or dementia
3. If the lifestyle changes were fun and enjoyable
4. If the lifestyle changes were affordable
5. If my relatives or friends developed memory impairment or dementia
6. If I received personal specific advice about what I should do (e.g. from my doctor
7. If I had support/ motivation from my friends/family
8. If the lifestyle changes were proven to be beneficial for brain health
9. Nothing would motivate me
10. Nothing would motivate me, I believe my brain health is already optimal
11. Other (Please specify) [Free text]

**5.6** **What could prevent you from improving your lifestyle to improve brain health?**[SELECT **UP TO 3 OPTIONS** THAT YOU CONSIDER TO BE THE **MOST IMPORTANT**. PLEASE SELECT **AT LEAST ONE** OPTION].

1. Lack of time
2. If I had to give up foods I like
3. If I had to start doing activities that I do not enjoy
4. Lack of motivation
5. Lack of information about what to do
6. If I had to make changes by myself/alone
7. If making changes was expensive
8. If I cannot be sure that the changes help
9. Current health problems
10. I feel no need to do anything
11. Other [*Please specify in free text*]

_________________________________________________________________________

**Section 6. In this section, we would like to ask you a few questions about dementia that affects many older people in our society.**

- 1. **Have you ever personally known anyone with dementia or have it yourself?**
- No, I don’t know anyone who has or had, dementia
- Yes, my job involves / involved working with people who have dementia
- Yes, I have dementia myself
- Yes, my partner or a member of my family (e.g. parents)
- Yes, a friend(s) I know fairly well
- Yes, a friend(s) or acquaintance(s) I know less well
- Yes, a colleague / someone at my work
- Yes, someone else
- Not sure

**Now we would like to better understand your knowledge and beliefs about dementia.**

- 1. **We would like to show you some statements and ask you to tell us to what extent you agree or disagree with each.**

| **Statement** | **True** | **False** | **I don’t know** |
| --- | --- | --- | --- |
| **6.2.1** My knowledge surrounding dementia is good  (Please select one answer in response to the statement) |  |  |  |
| **6.2.2** Dementia is a disease of the brain  (Please select one answer in response to the statement) |  |  |  |
| **6.2.3** Dementia is a mental illness  (Please select one answer in response to the statement) |  |  |  |
| **6.2.4** Dementia is part of the normal process of ageing  (Please select one answer in response to the statement) |  |  |  |
| **6.2.5** Dementia is another term for Alzheimer’s disease  (Please select one answer in response to the statement) |  |  |  |
| **6.2.6** There are drug treatments that help with dementia  (Please select one answer in response to the statement) |  |  |  |
| **6.2.7** There are many different kinds of dementia  (Please select one answer in response to the statement) |  |  |  |
| **6.2.8** Dementia can be cured  (Please select one answer in response to the statement) |  |  |  |

**_________________________________________________________________________**

**Section 7. This is the last section of the survey that asks some general questions about you.**

**7.1 These next four questions are about your feelings and thoughts in the last month. For each statement, please indicate how often you felt or thought a certain way.**

(Please select one answer in response to each statement)

|  | Statement | **Never** | **Almost Never** | **Sometimes** | **Fairly Often** | **Very Often** |
| --- | --- | --- | --- | --- | --- | --- |
| **7.1.1** | In the last month, how often have you felt that you were unable to control the important things in your life? |  |  |  |  |  |
| **7.1.2** | In the last month, how often have you felt confident about your ability to handle your personal problems? |  |  |  |  |  |
| **7.1.3** | In the last month, how often have you felt that things were going your way? |  |  |  |  |  |
| **7.1.4** | In the last month, how often have you felt difficulties were piling up so high that you could not overcome them? |  |  |  |  |  |

**The next few questions are about your general information.**

- 1. **What is your current relationship status?**
     (Please select one answer)
- Single
- Married and living with husband/wife
- Civil partnership (legally registered)
- Married and separated from husband/wife
- Divorced
- Widowed
- In a relationship
- I’d prefer not to answer this question
  1. **Would you describe the place where you live as...**(Please select one answer)
- A big city
- The suburbs or outskirts of a big city
- A small city or town
- A country village
- A farm or home in the country
  1. **Finally, has completing this survey made you think more about your own brain health?**(Please select one answer)
- Yes
  (Please enter any feedback/comments to describe how this survey has affected you)
- No
  1. **We are interested to know how this survey has affected your views about your own brain health.** **[Question only displayed if Q7.4 = Yes]

     Please enter any feedback/comments in the free text box below to describe this.** [Free text entry]

_________________________________________________________________________

**Optional section. COVID-19**

**Thank you for completing our survey.**

**We know that at the moment, your lifestyle choices might look a little different due to the ongoing COVID-19 pandemic.**

1. **To understand this further, we would be grateful if you could spare a few more minutes to answer questions relating to COVID-19, brain health and lifestyle factors.
   [If response = No, skip to end of survey]**

- Yes, I am willing to answer a few more questions relating to COVID-19, brain health and lifestyle factors
- No, I don't want to answer any more questions
  1. **Do you feel impacted by COVID-19?
     [If response = No, skip to end of survey]**
- Yes
- No
  1. **Have you had a test to check for COVID-19?**
- Yes
- No
  - 1. **What were the results of your COVID-19 test?
       [Question only displayed if Q8.2 = Yes]**
- Positive
- Negative
- Borderline/ Unclear/ Inconclusive/ Void
  - 1. **Have you received treatment for COVID-19?
       [Question only displayed if Q8.2.1 = Positive]**
- Yes
- No
  1. **Has the ongoing COVID-19 pandemic impacted your brain health?
     The term ‘brain health’ is a person’s ability to function well in daily life and work.
     This involves the brain’s capability to remember, comprehend and learn information, and the ability to think strategically to make everyday decisions.**
- Yes
- No
  - 1. **As a result, do you feel that your brain health has improved or deteriorated?
       [Question only displayed if Q8.3 = Yes]**
- Improved
- Deteriorated
- I’m not sure
  - 1. **We are interested to know why your brain health might have deteriorated during COVID-19. Please provide a reason for this using the free text box below. We'd kindly ask you try to be as open and as honest as possible with your answers.
       [Question only displayed if Q8.5 = Deteriorated]**
- [Free text entry]
  1. **Has the ongoing COVID-19 pandemic impacted your dietary habits?**
- Yes
- No
  - 1. **As a result, do you feel that your dietary habits have improved or deteriorated?
       [Question only displayed if Q8.4 = Yes]**
- Improved
- Deteriorated
- I’m not sure
  - 1. **We are interested to know why your dietary habits might have deteriorated during the COVID-19 pandemic. Please provide a reason for this using the free text box below. We'd kindly ask you try to be as open and as honest as possible with your answers.**

**[Question only displayed if Q8.4.1 = Deteriorated]**

**Displayed after completion of survey:**

Your brain healthy lifestyle score:

Living a healthy lifestyle can help you to keep your brain healthy, both now and in the future.

We considered some of your lifestyle factors to create your brain healthy lifestyle score.

You scored [Display participants score] out of 30.

If you would like to learn more about ways to keep your brain healthy, we have plenty of information and top tips to help.

Please click here to access our fact sheet.

PLEASE NOTE: This score does not provide medical advice and is intended for information purposes only. It should not be used as a substitute for professional medical advice, diagnosis or treatment. If you are concerned about your own health, you should contact a medical health professional to discuss this.
