# Supplementary material for: Awareness, facilitators, barriers, and behaviours surrounding brain health: a large-scale cross-sectional survey of adults across UK and Ireland
Source: BMC Public Health. 2025 Oct 1;25:3279. doi: 10.1186/s12889-025-24175-0 (PMC12487460; doi:10.1186/s12889-025-24175-0)
Supplement: Supplementary file 2 — Supplementary Material 2 [file 12889_2025_24175_MOESM2_ESM.docx]

Table S1. Overview of listed barriers and motivations and mapping to behaviour change frameworks

|  | **Options** | **TDF Domain** | **COM-B Domain** | **COM-B Construct** | **Respondents selection,**  **n (%)** |
| --- | --- | --- | --- | --- | --- |
| **Barriers** | If I had to start doing activities that I do not enjoy | Emotion | Automatic motivation | Motivation | 2639 (44.0) |
|  | If I had to give up foods I like | Emotion | Automatic motivation | Motivation | 1221 (20.4) |
|  | Lack of motivation | Optimism | Reflective motivation | Motivation | 1965 (32.8) |
|  | I feel no need to do anything | Optimism | Reflective motivation | Motivation | 272 (4.5) |
|  | If I cannot be sure that the changes help | Beliefs about capabilities | Reflective motivation | Motivation | 1611 (26.9) |
|  | If making changes was expensive | Environmental context and resources | Physical opportunity | Opportunity | 1220 (20.4) |
|  | Lack of time | Environmental context and resources | Physical opportunity | Opportunity | 989 (16.5) |
|  | If I had to make changes by myself/alone | Social influences | Social opportunity | Opportunity | 1141 (19.0) |
|  | Current health problems | Skills | Physical capability | Capability | 850 (14.2) |
|  | Lack of information about what to do | Knowledge | Psychological capability | Capability | 1626 (27.1) |
|  | Other | - | - | - | 222 (3.7) |
| **Facilitators** | If the lifestyle changes were fun and enjoyable | Emotion | Automatic motivation | Motivation | 1754 (29.1) |
|  | Nothing would motivate me, I believe my brain health is already optimal | Optimism | Reflective motivation | Motivation | 84 (1.4) |
|  | Nothing would motivate me | Beliefs about capabilities | Reflective motivation | Motivation | 21 (0.3) |
|  | If the lifestyle changes were affordable | Environmental context and resources | Physical opportunity | Opportunity | 580 (9.6) |
|  | If I received personal specific advice about what I should do | Social influences/ Knowledge | Social opportunity | Opportunity | 3068 (50.9) |
|  | If my relatives or friends developed memory impairment or dementia | Social influences | Social opportunity | Opportunity | 1138 (18.9) |
|  | If I had support/motivation from my friends/family | Social influences | Social opportunity | Opportunity | 653 (10.8) |
|  | If I noticed problems with my brain health | Memory, attention and decision processes | Psychological capability | Capability | 4235 (70.3) |
|  | If I had been diagnosed with memory impairment or dementia | Memory, attention and decision processes | Psychological capability | Capability | 2391 (39.7) |
|  | If the lifestyle changes were proven to be beneficial for brain health | Knowledge | Psychological capability | Capability | 1822 (30.2) |
|  | Other | - | - | - | 103 (1.7) |

TDF = Theoretical Domain Framework; COM-B = Capability, opportunity, and motivation for behaviour

Table S2. Differences between age categories in brain health beliefs

| **Brain Health Belief Statement** | **Response** | **Age Category** | | | | ***χ2*** |
| --- | --- | --- | --- | --- | --- | --- |
|  |  | **40-49** | **50-65** | **66-74** | **75+** |  |
| *I believe I am likely to experience poor brain health in the future* | Disagree | 102 (16.8) | 527 (18.7) | 477 (24.4) | 201 (27.6) | χ2(6) = 74.483, p <0.001, Cramer’s V = 0.083 |
|  | Not Sure | 206 (34.0) | 1114 (39.4) | 785 (40.1) | 282 (38.8) |  |
|  | Agree | 298 (49.2) | 1184 (41.9) | 696 (35.5) | 244 (33.6) |  |
| *There is a strong possibility that my brain health will decline in the next 10 years* | Disagree | 133 (21.9) | 430 (15.2) | 267 (13.6) | 77 (10.6) | χ2(6) = 49.745, p <0.001,  Cramer’s V = 0.072 |
|  | Not Sure | 186 (30.7) | 785 (27.8) | 520 (26.5) | 223 (30.6) |  |
|  | Agree | 287 (47.4) | 1612 (57.0) | 1173 (59.8) | 428 (58.8) |  |
| *The thought of my brain health declining scares me* | Disagree | 16 (2.6) | 84 (3.0) | 74 (3.8) | 38 (5.2) | χ2(6) = 36.913, p <0.001  Cramer’s V = 0.061 |
|  | Not Sure | 47 (7.8) | 241 (8.5) | 209 (10.7) | 102 (14.0) |  |
|  | Agree | 543 (89.6) | 2497 (88.5) | 1674 (85.5) | 588 (80.8) |  |
| *My feelings about myself would change if my brain health declined* | Disagree | 26 (4.3) | 117 (4.1) | 110 (5.6) | 49 (6.7) | χ2(6) = 45.051, p <0.001  Cramer’s V = 0.067 |
|  | Not Sure | 108 (17.8) | 597 (21.1) | 480 (24.5) | 206 (28.3) |  |
|  | Agree | 472 (77.9) | 2111 (74.7) | 1369 (69.9) | 472 (64.9) |  |
| *When I think about the possibility of my brain health declining, my heart beats* | Disagree | 182 (30.0) | 977 (34.6) | 758 (38.7) | 319 (43.8) | χ2(6) = 82.377, p <0.001, Cramer’s V = 0.090 |
|  | Not Sure | 186 (30.7) | 920 (32.5) | 680 (34.7) | 261 (35.9) |  |
|  | Agree | 238 (39.3) | 930 (32.9) | 523 (26.7) | 148 (20.3) |  |

Table S3. Differences between education categories in brain health beliefs

| **Brain Health Belief Statement** | **Response** | **Education Level** | | | | ***χ2*** |
| --- | --- | --- | --- | --- | --- | --- |
|  |  | **Primary** | **Secondary** | **Tertiary** | **Degree or above** |  |
| *I believe I am likely to experience poor brain health in the future* | Disagree | 28 (26.4) | 223 (21.5) | 200 (21.1) | 844 (21.2) | χ2(6) = 1.849, p >0.05, Cramer’s V = 0.017 |
|  | Not Sure | 38 (35.8) | 406 (39.2) | 367 (38.8) | 1554 (39.1) |  |
|  | Agree | 40 (37.7) | 406 (39.2) | 380 (40.1) | 1578 (39.7) |  |
| *There is a strong possibility that my brain health will decline in the next 10 years* | Disagree | 13 (12.1) | 137 (13.2) | 127 (13.4) | 624 (15.7) | χ2(6) = 9.013, p >0.05 |
|  | Not Sure | 33 (30.8) | 297 (28.7) | 250 (26.4) | 1114 (28.0) |  |
|  | Agree | 61 (57.0) | 601 (58.1) | 570 (60.2) | 2241 (56.3) |  |
| *The thought of my brain health declining scares me* | Disagree | 6 (5.7) | 52 (5.0) | 30 (3.2) | 123 (3.1) | χ2(6) = 12.372, p >0.05 |
|  | Not Sure | 11 (10.4) | 108 (10.4) | 91 (9.6) | 374 (9.4) |  |
|  | Agree | 89 (84.0) | 875 (84.5) | 824 (87.2) | 3477 (87.5) |  |
| *My feelings about myself would change if my brain health declined* | Disagree | 8 (7.5) | 57 (5.5) | 39 (4.1) | 197 (5.0) | χ2(6) = 6.477, p >0.05 |
|  | Not Sure | 18 (16.8) | 243 (23.5) | 225 (23.8) | 887 (22.3) |  |
|  | Agree | 81 (75.7) | 735 (71.0) | 683 (72.1) | 2891 (72.7) |  |
| *When I think about the possibility of my brain health declining, my heart beats* | Disagree | 27 (25.2) | 349 (33.7) | 296 (31.3) | 1552 (39.0) | χ2(6) = 31.435, p <0.001, Cramer’s V = 0.051 |
|  | Not Sure | 42 (39.3) | 358 (34.6) | 348 (36.7) | 1271 (31.9) |  |
|  | Agree | 38 (35.5) | 328 (31.7) | 303 (32.0) | 1157 (29.1) |  |

Table S4. Differences between country categories in brain health beliefs

| **Brain Health Belief Statement** | **Response** | **Country** | | | | ***χ2*** |
| --- | --- | --- | --- | --- | --- | --- |
|  |  | **Northern Ireland** | **Republic of Ireland** | **Scotland** | **England and Wales** |  |
| *I believe I am likely to experience poor brain health in the future* | Disagree | 142 (22.0) | 420 (29.2) | 147 (20.4) | 598 (18.1) | χ2(6) = 113.243, p <0.001, Cramer’s V = 0.091 |
|  | Not Sure | 267 (41.4) | 584 (40.6) | 286 (39.8) | 1250 (37.7) |  |
|  | Agree | 236 (36.6) | 435 (30.2) | 286 (39.8) | 1465 (44.2) |  |
| *There is a strong possibility that my brain health will decline in the next 10 years* | Disagree | 94 (14.6) | 305 (21.2) | 111 (15.4) | 397 (12.0) | χ2(6) = 104.025, p <0.001,  Cramer’s V = 0.087 |
|  | Not Sure | 193 (29.9) | 451 (31.3) | 199 (27.7) | 871 (26.3) |  |
|  | Agree | 358 (55.5) | 684 (47.5) | 409 (56.9) | 2049 (61.8) |  |
| *The thought of my brain health declining scares me* | Disagree | 21 (3.3) | 60 (4.2) | 21 (2.9) | 110 (3.3) | χ2(6) = 21.152, p = 0.002, Cramer’s V = 0.037 |
|  | Not Sure | 71 (11.0) | 106 (7.4) | 93 (13.0) | 329 (9.9) |  |
|  | Agree | 553 (85.7) | 1273 (88.5) | 603 (84.1) | 2873 (86.7) |  |
| *My feelings about myself would change if my brain health declined* | Disagree | 26 (4.0) | 85 (5.9) | 35 (4.9) | 156 (4.7) | χ2(6) = 23.936, p <0.001, Cramer’s V = 0.048 |
|  | Not Sure | 130 (20.2) | 274 (19.0) | 176 (24.5) | 811 (24.5) |  |
|  | Agree | 489 (75.8) | 1080 (75.1) | 508 (70.7) | 2347 (70.8) |  |
| *When I think about the possibility of my brain health declining, my heart beats* | Disagree | 189 (29.3) | 456 (31.7) | 268 (37.3) | 1323 (39.9) | χ2(6) = 93.427, p <0.001, Cramer’s V = 0.085 |
|  | Not Sure | 218 (33.8) | 440 (30.6) | 249 (34.6) | 1140 (34.4) |  |
|  | Agree | 238 (36.9) | 544 (37.8) | 202 (28.1) | 855 (25.8) |  |

Table S5. Frequency of consumption per each component of the MIND Score

| **MIND Component** | **0 Servings*** | **1 Serving** | **2 Servings** | **3 Servings** | **4 Servings** | **5 Servings** | **6 Servings** | **7 Servings or More**** |
| --- | --- | --- | --- | --- | --- | --- | --- | --- |
| ***Whole grains (p/d)*** | 473 (7.4) | 2196 (34.3) | 2545 (39.7) | 901 (14.1) | 230 (3.6) | 38 (0.6) | 14 (0.2) | 13 (0.2) |
| ***Butter or margarine (p/d)*** | 1043 (16.3) | 2241 (35.0) | 1768 (27.6) | 775 (12.1) | 403 (6.3) | 100 (1.6) | 46 (0.7) | 34 (0.5) |
| ***Green leafy vegetables (p/w)*** | 275 (4.3) | 378 (5.9) | 735 (11.5) | 1021 (15.9) | 960 (15.0) | 966 (15.1) | 510 (8.0) | 1565 (24.4) |
| ***Other vegetables (p/w)*** | 71 (1.1) | 149 (2.3) | 317 (4.9) | 702 (11.0) | 813 (12.7) | 967 (15.1) | 647 (10.1) | 935 (14.6) |
| ***Red or processed meat (p/w)*** | 1012 (15.8) | 930 (14.5) | 1217 (19.0) | 1206 (18.8) | 925 (14.4) | 634 (9.9) | 244 (3.8) | 242 (3.8) |
| ***Fish or shellfish per week (p/w)*** | 1347 (21.0) | 1596 (24.9) | 1895 (29.6) | 960 (15.0) | 384 (6.0) | 152 (2.4) | 39 (0.6) | 37 (0.6) |
| ***Chicken or turkey (non-fried) (p/w)*** | 1145 (17.9) | 1199 (18.7) | 1976 (30.8) | 1287 (20.1) | 546 (8.5) | 184 (2.9) | 36 (0.6) | 37 (0.6) |
| ***Beans and pulses (p/w)*** | 586 (9.1) | 921 (14.4) | 1704 (26.6) | 1463 (22.8) | 822 (12.8) | 465 (7.3) | 165 (2.6) | 284 (4.4) |
| ***Cheese (p/w)*** | 872 (13.6) | 675 (10.5) | 1198 (18.7) | 1265 (19.7) | 937 (14.6) | 642 (10.0) | 323 (5.0) | 498 (7.8) |
| ***Natural/raw nuts (p/w)*** | 2634 (41.1) | 905 (14.1) | 760 (11.9) | 579 (9.0) | 422 (6.6) | 394 (6.1) | 184 (2.9) | 532 (8.3) |
| ***Berries (p/w)*** | 1314 (20.5) | 902 (14.1) | 916 (14.3) | 767 (12.0) | 606 (9.5) | 560 (8.7) | 296 (4.6) | 1049 (16.4) |
| ***Wine (p/w)*** | 2845 (44.3) | 318 (5.0) | 462 (7.2) | 460 (7.2) | 497 (7.8) | 394 (6.1) | 499 (7.8) | 935 (14.6) |
| ***Dessert/sweet foods (p/w)*** | 627 (9.8) | 457 (7.1) | 778 (12.1) | 823 (12.8) | 747 (11.7) | 727 (11.3) | 577 (9.0) | 1674 (26.1) |
| ***Take-away food (p/w)*** | 4996 (77.9) | 1134 (17.7) | 226 (3.5) | 34 (0.5) | 8 (0.1) | 7 (0.1) | 3 (0.0) | 2 (0.0) |

*** "0 Servings" response was used for categories of whole-grains, butter/margarine, red/processed meat; “I do not drink alcohol” and “Less than 1 glass per week” were combined for the category of wine, and "Less than 1 Serving" for all remaining categories.**

**** "7 Servings or More" for the whole-grains and butter/margarine categories and "7 Servings or More" for other categories.**

|  |  | **Age Category** | | | | ***χ2*** |
| --- | --- | --- | --- | --- | --- | --- |
|  |  | **40-49** | **50-65** | **66-74** | **75+** |  |
|  |  | n selected (column %) | n selected (column %) | n selected (column %) | n selected (column %) |  |
| *Potential Barriers* | If I had to start doing activities that I do not enjoy | 211 (35.3) | 1117 (42.6) | 932 (48.7) | 319 (44.6) | χ2(3) = 37.829, p <0.001, Cramer’s V = 0.076 |
|  | Lack of motivation | 233 (39.0) | 941 (34.0) | 585 (30.5) | 206 (28.8) | χ2(3) = 21.745, p <0.001, Cramer’s V = 0.056 |
|  | Lack of information about what to do | 125 (20.9) | 723 (26.1) | 566 (29.6) | 212 (29.7) | χ2(3) = 21.104, p <0.001, Cramer’s V = 0.053 |
|  | If I cannot be sure that the changes help | 112 (18.7) | 686 (24.8) | 579 (30.2) | 234 (32.7) | χ2(3) = 49.704, p <0.001, Cramer’s V = 0.101 |
|  | If I had to give up foods I like | 154 (25.8) | 565 (20.4) | 382 (19.9) | 120 (16.8) | χ2(3) = 16.567, p <0.001, Cramer’s V = 0.054 |
|  | If making changes was expensive | 157 (14.2) | 613 (22.2) | 340 (17.8) | 110 (15.4) | χ2(3) = 37.293, p <0.001, Cramer’s V = 0.101 |
|  | If I had to make changes by myself/alone | 85 (14.2) | 493 (17.8) | 409 (21.4) | 154 (21.5) | χ2(3) = 21.263, p <0.001, Cramer’s V = 0.062 |
|  | Lack of time | 275 (46.0) | 564 (20.4) | 118 (6.2) | 32 (4.5) | χ2(3) = 631.362, p <0.001, Cramer’s V = 0.410 |
|  | Current health problems | 60 (10.0) | 324 (11.7) | 312 (16.3) | 154 (21.5) | χ2(3) = 61.108, p <0.001, Cramer’s V = 0.132 |
|  | I feel no need to do anything | 13 (2.2) | 108 (3.9) | 94 (4.9) | 57 (8.0) | χ2(3) = 30.348, p <0.001, Cramer’s V = 0.204 |
|  | Other | 24 (4.0) | 119 (4.3) | 59 (3.1) | 20 (2.8) | χ2(3) = 6.669, p = 0.083 |
| *Potential Motivations* | If I received personal specific advice about what I should do | 272 (45.2) | 1337 (48) | 1049 (54.5) | 410 (57.3) | χ2(3) = 38.870, p <0.001, Cramer’s V = 0.064 |
|  | If the lifestyle changes were proven to be beneficial for brain health | 156 (25.9) | 801 (28.8) | 633 (32.9) | 232 (32.4) | χ2(3) = 16.180, p = 0.001, Cramer’s V = 0.041 |
|  | If the lifestyle changes were fun and enjoyable | 218 (36.2) | 218 (36.2) | 806 (28.9) | 551 (28.6) | χ2(3) = 20.748, p <0.001, Cramer’s V = 0.074 |
|  | If my relatives or friends developed memory impairment or dementia | 146 (24.3) | 146 (24.3) | 605 (21.7) | 294 (15.3) | χ2(3) = 58.590, p <0.001, Cramer’s V = 0.120 |
|  | If the lifestyle changes were affordable | 87 (14.5) | 87 (14.5) | 281 (10.1) | 167 (8.7) | χ2(3) = 27.962, p <0.001, Cramer’s V = 0.134 |
|  | Other | 13 (2.2) | 13 (2.2) | 62 (2.2) | 21 (1.1) | χ2(3) = 11.821, p = 0.008, Cramer’s V = 0.165 |
|  | If I noticed problems with my brain health | 411 (68.3) | 411 (68.3) | 1937 (69.6) | 1380 (71.7) | χ2(3) = 3.735, p = 0.292 |
|  | If I had been diagnosed with memory impairment or dementia | 230 (38.2) | 230 (38.2) | 1109 (46.4) | 785 (40.8) | χ2(3) = 0.371, p = 0.371 |
|  | If I had support/motivation from my friends/family | 78 (13.0) | 78 (13.0) | 311 (11.2) | 194 (10.1) | χ2(3) = 0.165, p = 0.165 |
|  | Nothing would motivate me, I believe my brain health is already optimal | 5 (0.8) | 5 (0.8) | 33 (1.2) | 32 (1.7) | χ2(3) = 4.935, p = 0.177 |

Table S6. Differences between age categories in motivations for, and barriers to the promotion of brain health

Table S7. Tests of chi-squared to explore differences between education categories in motivations for, and barriers to the promotion of brain health

|  |  | **Education category** | | | | ***χ2*** |
| --- | --- | --- | --- | --- | --- | --- |
|  |  | **Primary** | **Secondary** | **Tertiary** | **Degree level or above** |  |
|  |  | n selected (column %) | n selected (column %) | n selected (column %) | n selected (column %) |  |
| *Potential Barriers* | If I had to start doing activities that I do not enjoy | 31 (30.4) | 392 (38.9) | 407 (44.3) | 1417 (45.9) | χ2(3) = 23.766, p <0.001, Cramer’s V = 0.059 |
|  | Lack of motivation | 35 (34.3) | 374 (37.1) | 331 (36) | 1209 (30.9) | χ2(3) = 19.625, p < 0.001, Cramer’s V = 0.058 |
|  | If I cannot be sure that the changes help | 20 (19.6) | 226 (22.4) | 235 (25.6) | 1124 (28.7) | χ2(3) = 20.149, p < 0.001, Cramer’s V = 0.065 |
|  | If I had to give up foods I like | 18 (17.6) | 164 (16.3) | 181 (19.7) | 851 (21.7) | χ2(3) = 15.512, p = 0.001, Cramer’s V = 0.064 |
|  | If making changes was expensive | 31 (30.4) | 252 (25.0) | 219 (23.8) | 714 (18.2) | χ2(3) = 37.430, p <0.001, Cramer’s V = 0.079 |
|  | Lack of time | 7 (6.9) | 111 (11.0) | 132 (14.4) | 737 (18.8) | χ2(3) = 46.862, p <0.001, Cramer’s V = 0.125 |
|  | Current health problems | 20 (19.6) | 177 (17.6) | 133 (14.5) | 500 (12.8) | χ2(3) = 18.505, p <0.001, Cramer’s V = 0.078 |
|  | If I had to make changes by myself/alone | 22 (33.3) | 219 (21.7) | 168 (18.3) | 721 (18.4) | χ2(3) = 6.564, p = 0.087 |
|  | Lack of information about what to do | 34 (33.3) | 291 (28.9) | 255 (27.7) | 1032 (26.4) | χ2(3) = 4.950, p = 0.176 |
| *Potential Motivations* | If the lifestyle changes were proven to be beneficial for brain health | 23 (22.3) | 290 (28.6) | 262 (28.4) | 1233 (31.3) | χ2(3) = 8.028, p = 0.045, Cramer’s V = 0.041 |
|  | If the lifestyle changes were fun and enjoyable | 22 (21.4) | 251 (24.8) | 266 (28.8) | 1199 (30.5) | χ2(3) = 15.714, p = 0.001, Cramer’s V = 0.069 |
|  | If the lifestyle changes were affordable | 14 (13.6) | 120 (11.8) | 116 (12.6) | 323 (8.2) | χ2(3) =25.940, p <0.001, Cramer’s V = 0.120 |
|  | If I noticed problems with my brain health | 71 (68.9) | 716 (70.7) | 669 (72.4) | 2746 (69.7) | χ2(3) = 2.693, p = 0.441 |
|  | If I received personal specific advice about what I should do | 54 (52.4) | 526 (51.9) | 464 (50.2) | 2009 (51.0) | χ2(3) = 0.644, p = 0.886 |
|  | If I had been diagnosed with memory impairment or dementia | 32 (31.1) | 386 (38.1) | 371 (40.2) | 1589 (40.4) | χ2(3) = 5.057, p = 0.168 |
|  | If my relatives or friends developed memory impairment or dementia | 20 (18.4) | 191 (18.9) | 180 (19.5) | 735 (65.3) | χ2(3) = 0.346, p = 0.951 |
|  | If I had support/motivation from my friends/family | 10 (9.7) | 92 (9.1) | 90 (9.7) | 452 (11.5) | χ2(3) = 6.211, p = 0.102 |

Table S8. Tests of chi-squared to explore differences between countries in motivations for, and barriers to the promotion of brain health

|  |  | **Country of residence category** | | | | ***χ2*** |
| --- | --- | --- | --- | --- | --- | --- |
|  |  | **Northern Ireland** | **Republic of Ireland** | **Scotland** | **England and Wales** |  |
|  |  | n selected (column %) | n selected (column %) | n selected (column %) | n selected (column %) |  |
| *Potential Barriers* | If I had to start doing activities that I do not enjoy | 269 (42.5) | 534 (38.3) | 285 (40.7) | 1551 (47.5) | χ2(3) = 37.910, p <0.001, Cramer’s V = 0.071 |
|  | Lack of motivation | 249 (39.3) | 517 (37.1) | 249 (35.5) | 950 (29.1) | χ2(3) = 46.924, p <0.001, Cramer’s V = 0.077 |
|  | If I cannot be sure that the changes help | 146 (23.1) | 306 (22.0) | 177 (25.2) | 982 (30.1) | χ2(3) = 39.536, p <0.001, Cramer’s V = 0.102 |
|  | If I had to make changes by myself/alone | 143 (22.6) | 235 (16.9) | 137 (19.5) | 626 (19.2) | χ2(3) = 9.581, p = 0.022, Cramer’s V = 0.053 |
|  | If I had to give up foods I like | 133 (21.0) | 269 (19.3) | 141 (20.1) | 678 (20.8) | χ2(3) = 1.447, p = 0.694 |
|  | If making changes was expensive | 122 (19.3) | 266 (19.1) | 134 (19.1) | 698 (21.4) | χ2(3) = 4.451, p = 0.209 |
|  | Lack of time | 145 (22.9) | 251 (18.0) | 97 (13.8) | 496 (15.2) | χ2(3) = 28.917, p <0.001, Cramer’s V = 0.098 |
|  | Current health problems | 83 (13.1) | 171 (12.3) | 108 (15.4) | 488 (14.9) | χ2(3) = 7.160, p = 0.67 |
|  | Lack of information about what to do | 158 (25.0) | 392 (28.1) | 169 (24.1) | 907 (27.8) | χ2(3) = 6.126, p = 0.106 |
|  | I feel no need to do anything | 29 (4.6) | 65 (4.7) | 33 (4.7) | 145 (4.4) | χ2(3) = 0.177, p = 0.981 |
|  | Other | 16 (2.5) | 51 (3.7) | 28 (4.0) | 127 (3.9) | χ2(3) = 2.937, p = 0.401 |
| *Potential Motivations* | If the lifestyle changes were proven to be beneficial for brain health | 200 (31.4) | 399 (28.5) | 183 (25.9) | 1040 (31.7) | χ2(3) = 11.946, p = 0.008, Cramer’s V = 0.060 |
|  | If the lifestyle changes were affordable | 54 (8.5) | 106 (7.6) | 72 (10.2) | 348 (10.6) | χ2(3) = 11.529, p = 0.009, Cramer’s V = 0.089 |
|  | If I noticed problems with my brain health | 475 (74.7) | 1009 (72.1) | 501 (70.9) | 2250 (68.5) | χ2(3) = 13.189, p = 0.004, Cramer’s V = 0.038 |
|  | If I had been diagnosed with memory impairment or dementia | 234 (36.8) | 477 (34.1) | 251 (35.5) | 1429 (43.5) | χ2(3) = 45.812, p <0.001, Cramer’s V = 0.088 |
|  | If my relatives or friends developed memory impairment or dementia | 144 (22.6) | 299 (21.4) | 131 (18.5) | 564 (17.2) | χ2(3) = 17.822, p <0.001, Cramer’s V = 0.077 |
|  | Other | 9 (1.4) | 13 (0.9) | 26 (3.7) | 55 (1.7) | χ2(3) = 21.740, p <0.001, Cramer’s V = 0.126 |
|  | If the lifestyle changes were fun and enjoyable | 190 (29.9) | 385 (27.5) | 204 (28.9) | 975 (29.7) | χ2(3) = 2.479, p = 0.479 |
|  | If I received personal specific advice about what I should do | 326 (51.3) | 693 (49.5) | 369 (52.2) | 1680 (51.1) | χ2(3) = 1.680, p = 0.641 |
|  | If I had support/motivation from my friends/family | 67 (10.5) | 126 (9.0) | 86 (12.2) | 374 (11.4) | χ2(3) = 7.262, p = 0.064 |
|  | Nothing would motivate me, I believe my brain health is already optimal | 7 (1.1) | 15 (1.1) | 8 (1.1) | 54 (1.6) | χ2(3) = 3.305, p = 0.347 |

Figure S1. Relationship between respondents country and education.

Figure S2. Relationship between respondents country and age.

Figure S3. Relationship between respondents age and education.
